# Ambivalent Copper: Mechanistically Distinct Immune Effects Driving Innovation in Cancer Nanomedicine

**DOI:** 10.3390/pharmaceutics18010075

**Published:** 2026-01-07

**Authors:** Devon Heroux, Xu Xin Sun, Zeynab Nosrati, Marcel B. Bally

**Affiliations:** 1 Department of Basic and Translational Research, BC Cancer Research Institute, Vancouver, BC V5Z 0B4, Canadambally@bccrc.ca (M.B.B.); 2 Department of Pathology and Laboratory Medicine, University of British Columbia, Vancouver, BC V6T 1Z7, Canada; 3 Faculty of Medicine, University of British Columbia, Vancouver, BC V6T 1Z3, Canada; 4 Faculty of Pharmaceutical Sciences, University of British Columbia, Vancouver, BC V6T 1Z3, Canada

**Keywords:** copper, Cu, cancer, immunity, nanomedicines, ionophore, copper deficiency, immune cells, immunotherapy, immune checkpoint inhibitors, PD-1, PD-L1

## Abstract

Copper (Cu) is an essential element required by all living cells, where it supports critical enzymatic and signaling functions. In cancer, this balance is often disrupted, creating vulnerabilities that can be therapeutically exploited. Changes in Cu availability have been shown to influence key immunoregulatory pathways, including those involved in inflammation, cell death, and immune evasion. Notably, Cu can drive expression of programmed death ligand 1 (PD-L1), contributing to immunosuppression, while also promoting immunogenic cell death, which stimulates adaptive immune responses. These dual effects highlight the complexity and therapeutic potential of Cu-based interventions, particularly in the context of immune modulation and toxicity. This review argues that Cu-based nanomedicines can selectively deliver high concentrations of bioactive Cu to tumor cells, inducing cell death and triggering adaptive immune responses. We summarize current knowledge on Cu’s roles in cancer and immunity, emphasizing recent insights into how these intersect through Cu-mediated modulation of anticancer immune pathways. Finally, we explore the clinical potential of Cu-based nanomedicines to convert immunologically “cold” tumors into “hot” ones, thereby improving responses to immunotherapy. Realizing this potential will depend on the thoughtful integration of Cu delivery approaches with existing immunotherapeutic strategies.

## 1. Introduction

Transition metals have a variety of important roles in biological processes, with zinc (Zn), iron (Fe), manganese (Mn), cobalt (Co), and Cu being essential for human health [1]. While redox-inactive Zn signals through fluctuations of ion pools, the latter four metals function largely as static co-factors that drive redox catalysis through Fenton chemistry, although recently the concept of metalloallostery has emerged as a new paradigm of protein regulation through metal ions binding allosteric sites [2,3]. Approximately 30 enzymes utilize Cu as a co-factor, including cytochrome c oxidase, superoxide dismutase, and lysyl oxidase, which regulate the electron transport chain, free radical scavenging, and crosslinking of collagen, respectively [3].

Cu(II) has also been identified as a regulator of MEK1/2 and ULK1/2, resulting in MAPK activation and autophagy, which are frequently upregulated in cancer cells [4,5]. Accordingly, high levels of Cu are found in many tumor types where it is a driver of proliferation, angiogenesis, and metastasis, whereas a disruption of Cu homeostasis through either Cu deprivation or Cu excess results in cell death through inhibition of cuproplasia (Cu-dependent cell growth and proliferation) or activation of cell death pathways, including apoptosis, and caspase-independent cell death. The latter are related to the recently described cuproptosis cell death mechanism [6,7,8,9]. These observations highlight that Cu availability is tightly linked to tumor cell survival, stress responses, and vulnerability to therapeutic intervention.

These two strategies of Cu modulation have been studied clinically with varied success, with an important consideration being the effect on the immune system. While Cu deprivation results in a marked decrease in many aspects of the immune system, some data have demonstrated that Cu excess may enhance the activity of immune cells, an important consideration given the role of the immune system in antitumor responses. In recent years, the direct role of Cu in tumor immunity includes immune-activating properties through induction of immunogenic cell death and immune silencing via overexpression and stabilization of PD-L1 [10,11,12,13,14,15]. Notably, Cu ionophores such as disulfiram and its metabolite diethyldithiocarbamate have emerged as widely studied examples of this duality, linking intracellular Cu delivery to both immunogenic cell death and modulation of immune checkpoint pathways. The dynamic signaling role of Cu also results in modulation of pathways such as NF-κB and ALDH1, which leads to variable expression of immune signaling through cytokines and retinoic acid [16,17,18,19]. Together, these findings position Cu as a context-dependent regulator of tumor–immune interactions, capable of promoting either immune activation or immune suppression depending on cellular context and mode of Cu modulation.

**Figure 1:**
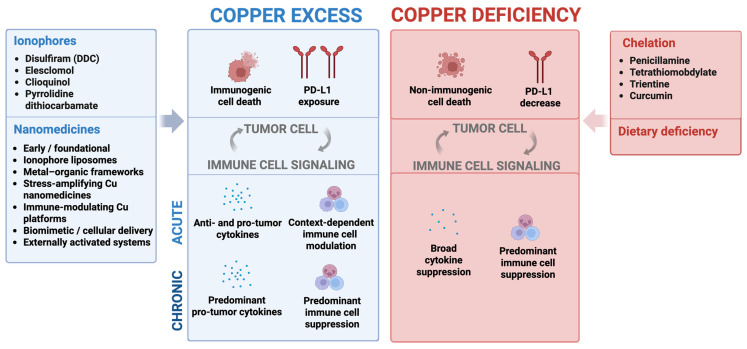
Schematic overview of the bidirectional effects of copper excess and copper deficiency on tumor–immune interactions. Copper availability shapes tumor cell stress responses and immune signaling in a context- and exposure-dependent manner. Acute copper excess, achieved through ionophores or copper-based nanomedicines, can induce immunogenic cell death and modulate PD-L1 exposure, leading to dynamic changes in cytokine production and immune cell activity that range from immune activation to immune suppression. With chronic exposure, copper excess may favor pro-tumor cytokine profiles and immune suppression. In contrast, copper deficiency induced by dietary restriction or chelation predominantly results in non-immunogenic cell death, reduced PD-L1 expression, broad cytokine suppression, and impaired immune cell function. Together, these opposing states highlight copper as a tunable regulator of tumor–immune crosstalk and underscore the importance of formulation, timing, and context when leveraging copper modulation for cancer immunotherapy.

The goal of this review is to discuss recent advances in the study of Cu in anticancer immunity, from a perspective of achieving improved treatment responses through Cu deprivation or Cu delivery. The effects of Cu modulation on immune cells, as well as the effect on tumor cells, are described by outlining several mechanisms of immune activation and/or immune silencing. Recent progress in the field of Cu nanomedicines as a potential way to deliver Cu-conjugated therapies has been summarized. Nanoformulations help to address concerns about Cu/Cu complex solubility while taking advantage of the potential tumor-targeting properties of nanoformulations. The conclusion highlights gaps in knowledge about Cu’s role in the immune system and its use to improve or inhibit treatment outcomes. This conclusion suggests that Cu delivery, immunogenic cell death, and Cu-mediated effects can all engender improved treatment outcomes when used in combination with existing immunotherapeutics. A schematic overview of these concepts is provided in Figure 1, illustrating the bidirectional immune effects of Cu and their therapeutic implications.

## 2. Cu’s Role in Cancer and Cancer Treatments

Cu is considered to be an important target in cancer due to its essential role in the cell and its dysregulation in many cancers [6]. Cu is an elemental nutrient and a key component of some critical enzymes involved in fundamental biological pathways [20]. In normal conditions, Cu levels in the body are controlled through a balance between absorption and distribution. In some cancers, alterations in Cu absorption, transport, metabolism, or excretion have led to higher Cu serum levels [21,22]. This may be due to higher demand for nutrients by cancer cells and this, in turn, is linked to an elevation of serum and tissue levels of Cu in various cancers including breast [23,24,25,26], ovarian [25,27,28,29], lung [30,31,32,33], colorectal [25,34,35,36], stomach [37,38], thyroid [39,40], acute leukemia [41,42], oral [43,44,45] and prostate [46,47] (Table 1). Moreover, various studies have confirmed that serum Cu level correlated with the grade of malignancy in some cancers and suggest serum Cu measurements could be used as a screening tool for cancer diagnosis/prognosis [48,49]. Ceruloplasmin, the major Cu-carrying protein in the blood, is another potential prognostic marker. Higher serum ceruloplasmin levels (4–8-fold over normal levels) have been reported in numerous cancers during tumor progression, which returns to normal during tumor regression [50,51]. With this in mind, it is important to consider how Cu might influence the various hallmarks of cancer [52,53,54]. In the context of this review, these cancer-associated functions of Cu are particularly relevant because they shape tumor stress responses, vascularization, and treatment sensitivity, all of which intersect with antitumor immunity.

### 2.1. Role of Cu in Tumor Angiogenesis

Angiogenesis is a normal physiological process in which new blood vessels are formed from the existing vasculature in developing or healing tissues. In addition, it plays a critical role in the growth of cancer as the newly formed blood vessels are needed to provide oxygen, nutrients, and other essential factors to the rapidly dividing cancer cells [55,56]. The tumor-associated blood vessels are, however, abnormal and lack a basement membrane and are tortuous, contributing to these blood vessels being “leaky” to serum. Tumor angiogenesis is regulated by many diverse factors. This can include hypoxia that can initiate the formation and release of vascular endothelial growth factor (VEGF), a potent mediator of blood vessel formation [57]. Findings have linked tumor angiogenesis with higher Cu and ceruloplasmin levels in serum [58,59]. Cu also has an essential role in the regulation of hypoxia-inducible factor-1 (HIF-1). Under hypoxic conditions, HIF-1 binds the VEGF gene promoter and promotes angiogenesis [60]. On the other hand, depletion of Cu using Cu chelation therapy has been shown to inhibit angiogenesis in a wide variety of cancers and cancer models [61,62]. PSP-2 is a Cu(I) selective chelator that has induced significant anti-angiogenic activity due to its ability to reduce intracellular Cu levels [63]. Because tumor angiogenesis influences immune cell trafficking, hypoxia, and therapeutic penetration, Cu-dependent regulation of angiogenic pathways has important implications for both immune surveillance and treatment response.

**Table 1:** Serum and tissue Cu levels in normal and cancer patients.

Cancer	Serum Cu Level (µg/dL)			Tissue Cu Level (µg/g)		
	Normal	Cancer		Normal	Cancer	
Breast	50.6 ± 12.8	105.6 ± 12.8	[24]	9.3 ± 2.3	21.0 ± 10.7	[26]
	98.8 ± 24.3	167.3 ± 37.9	[23]	1.58 ± 0.62	1.91 ± 0.56	[25]
Ovarian	106.73 ± 26.37	146 ± 24.78	[28]	0.3 ± 0.1	0.7 ± 0.3	[29]
	92.9	139.5	[64]	1.26 ± 0.45	2.16 ± 0.63	[25]
Lung	109.5 ± 5.39	122.9 ± 3.77	[33]	1.01 ± 0.02	1.52 ± 0.08	[30]
	128.5 ± 5.23	162.4 ± 8.18	[32]	5.08 ± 1.09	8.23 ± 4.88	[31]
Colon	152.08 ± 112.56	154.60 ± 91.71	[34]	1.53 ± 0.35	1.90 ± 0.6	[25]
	135.8 ± 30.5	138.6 ± 30.8	[36]	1.26 ± 0.37	1.47 ± 0.58	[35]
Stomach	143.03 ± 3.25	171.94 ± 7.27	[37]	1.1 ± 0.4	1.7 ± 0.4	[38]
Thyroid	105.87 ± 10.68	131.61 ± 33.9	[39]	4.23 ± 0.18	14.5 ± 2.6	[40]
Leukemia	86.7 ± 25.3	132.8 ± 50.6	[42]	15 ± 4 *	52 ± 16 *	[41]
Oral	124.83 ± 20.68	151.20 ± 11.20	[44]			
	114.20 ± 38.69	209.85 ± 160.28	[43]			
	105.5 + 18.81	141.99 ± 21.44	[45]			
Prostate	97 ± 22	169 ± 31	[47]			
	94.45 ± 34.37	100.31 32.38	[46]			

* μg/10^6^ cells.

### 2.2. Role of Cu in Tumor Metastasis

In addition to promoting tumor growth, Cu-dependent pathways play a central role in metastatic progression, with downstream consequences for tumor–immune interactions and treatment resistance. Cu ions appear to be required for the formation of pre-metastatic niches as well as the establishment of metastasis through Cu-binding proteins. Lysyl oxidase (LOX) and LOX-like (LOXL1–4) proteins are Cu-dependent metalloenzymes, secreted under hypoxic conditions by various cancers such as breast, pancreatic, hepatic, and colorectal cancers [65]. LOX family proteins are characterized by their Cu-binding sites at the C-terminal domain of the protein. The catalytic function of LOX is activated once it binds to Cu as a co-factor [66]. To support intracellular Cu delivery, the CTR1 transmembrane protein transports Cu ions into cells. Thereafter, metallochaperones like antioxidant protein 1 (ATOX1) mediate the delivery of Cu to the Menkes Cu-transporting ATPase (ATP7A and ATP7B) in the trans-Golgi network. ATP7A is responsible for pumping intracellular Cu to secreted LOX cuproenzymes. Subsequently, the Cu-loaded LOX mediates tumor progression through separate extracellular and intracellular mechanisms. Within extracellular mechanisms, LOX catalyzes the oxidation of lysine residues in elastin and collagen and forms a crosslinked elastin–collagen in the tumor microenvironment, which facilitates tumor growth and invasion [67,68]. It has been shown that silencing ATOX1 and ATP7A genes can inhibit the LOX activity, tumor growth, and metastasis in various models of lung, breast, and head and neck cancers [57,58,59]. In addition to this extracellular influence, LOX plays a key role in tumor cell migration and invasion. The by-product of LOX activation is hydrogen peroxide, which stimulates two key signaling molecules: focal adhesion kinase (FAK1) and proto-oncogene tyrosine-protein kinase (SRC). This promotes cell migration and metastasis [69,70]. Mediator of cell motility 1 (MEMO1) is another Cu-dependent enzyme involved in tumor metastasis. MEMO1 is upregulated in tumors, and its expression is correlated with cancer aggressiveness [71]. In mammary epithelial cells, MEMO1 mediates HER2-dependent cell migration through upregulation of IGF-IR/IRS1 signaling [72]. Recent studies have investigated MEMO1 inhibition as a potential therapeutic target for cancer treatment [73,74,75].

### 2.3. Role of Cu in Intrinsic and Acquired Chemotherapy Resistance

Platinum-based drugs are chemotherapeutic agents widely used for the treatment of solid tumors, including colon, breast, ovarian, bladder, brain, and non-small cell lung cancers [76,77,78,79,80,81]. The most common platinum agents are cisplatin, carboplatin, and oxaliplatin, which have unique roles in the management of individual cancers [82]. However, resistance to platinum-based drugs develops due to increases in DNA repair, reductions in cellular accumulation, and increases in drug inactivation [82]. Studies have shown that cell entry and subcellular distribution of platinum-containing drugs are mediated through transporter proteins (CTR1 and CTR2) and chaperones (ATOX1, ATP7A, and ATP7B), which, as indicated above, are also known to be involved in Cu homeostasis [83]. CTR1, the main Cu uptake transporter, regulates uptake and controls the accumulation and cytotoxic effect of platinum therapeutics. Studies have shown a good correlation between CTR1/CTR2 expression and chemoresistance to platinum-based drugs [27,84,85]. Elevated concentrations of Cu and platinum-based compounds have opposite effects on the regulation of CTR1 and CTR2. When there is an excess amount of Cu and platinum-containing drugs, CTR1 is downregulated through endocytosis and degradation, while CTR2 expression is increased [85]. Moreover, it has been shown that silencing of CTR2 leads to an increase in the cellular accumulation of cisplatin, and this has been proposed as a treatment to overcome platinum resistance [27]. Recent evidence demonstrates that higher expression of the Cu transporters ATP7A and ATP7B in different tumor types contributes to reduced sensitivity of platinum-based treatments due to an increase in drug efflux [75]. Several studies have investigated the inhibition of ATP7A and ATP7B as potential targets in cancer treatments. For example, silencing ATP7A and ATP7B expressions in ovarian cancer and breast cancers has improved platinum drug sensitivity [86,87,88,89]. Because these transporters regulate both Cu and platinum handling, their modulation represents a potential strategy to sensitize tumors to therapy while reshaping intracellular Cu signaling relevant to immune activation.

## 3. Cu Modulation as a Therapeutic Strategy in Cancer

Cu ions play essential roles in numerous biological processes, many of which are directly linked to cancer development and progression. Despite this, platinum-based compounds remain the only clinically approved class of metal-based anticancer agents. Therapies that modulate Cu homeostasis are already established in other contexts, such as Cu gluconate for deficiency and chelating agents like penicillamine or trientine for Wilson’s disease, but their potential in oncology has been largely overlooked. Given Cu’s ability to influence both tumor growth and stress-induced cell death, recent studies have focused on strategies that either elevate or deplete Cu to achieve therapeutic benefit. The following sections summarize these complementary approaches, Cu ionophores that deliver Cu into cells and Cu chelators that sequester it, and their emerging roles in cancer treatment. Where relevant, emphasis is placed on how these strategies influence tumor stress responses and immune signaling, rather than cytotoxicity alone.

### 3.1. Cu Ionophores

**Figure 2:**
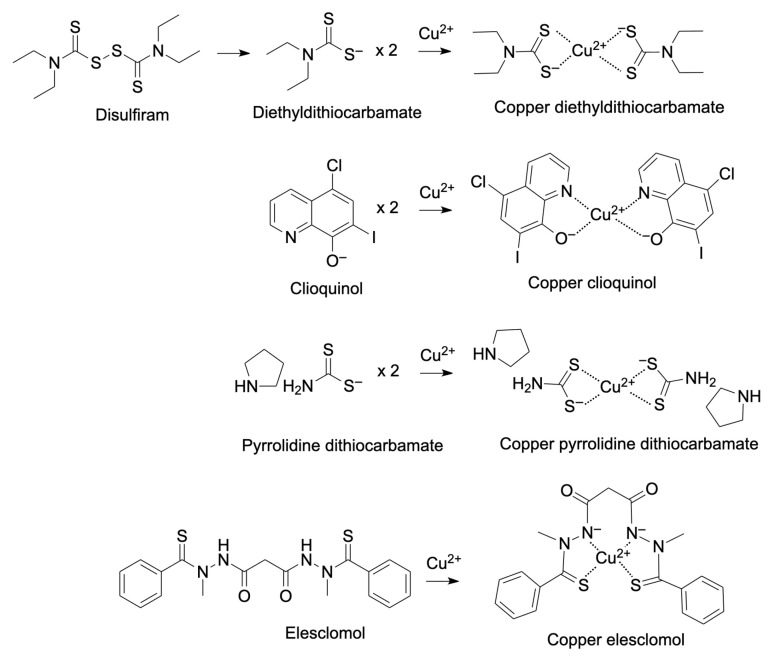
Representative copper-binding agents and their Cu(II) complexes. Disulfiram is metabolized to diethyldithiocarbamate (DDC), which chelates Cu^2+^ to form a 2:1 ligand–metal complex. Clioquinol and pyrrolidine dithiocarbamate similarly coordinate Cu^2+^ through bidentate donor atoms, while elesclomol forms a 1:1 Cu(II) complex. These Cu complexes form the basis of widely studied copper ionophore and delivery strategies discussed in this review.

Cu ionophores efficiently transport Cu across biological membranes, providing a payload of Cu that can selectively induce cuproptosis in cancer cells [9,90]. The role of Cu ionophores in cancer has been recently reviewed by Oliveri et al. [90], and will be briefly discussed here in the context of cancer immunity, an area of growing interest and attention. Table 2 identifies commonly used Cu ionophores and their known cellular and immunological effects, with structures and Cu coordination indicated in Figure 2. The most commonly used of these compounds in cancer is disulfiram (DSF), which is an anti-alcohol abuse drug that has shown potential as an anticancer agent since the 1970s, although efforts to use it clinically have not been successful [91]. This may be due to its rapid systemic elimination and its requirement for Cu to be active against cancer. This is where our lab became interested in the DSF metabolite diethyldithiocarbamate (DDC), a well-known Cu-binding agent [92,93,94]. When DSF is administered, it is converted in the blood to DDC, which may undergo oxidative biotransformation to diethylthiomethylcarbamate (Me-DTC) [95]. This compound acts as a suicide inhibitor for aldehyde dehydrogenases (ALDHs), although in the presence of Cu, DDC will form Cu(DDC)_2_, the complex responsible for DSF’s anticancer activity. This may be preferentially formed within cancer cells, which have a higher level of intracellular Cu, although most studies with DSF have been in combination with Cu, or as a soluble form of Cu(DDC)_2_ [91,96]. The mechanisms through which Cu ionophores can act against cancer or modulate immune responses are summarized in Table 2, but are further described in Section 5 of this review.

Novel small molecules have also been developed recently to expand the range of Cu ionophores available for targeted cancer therapy. For example, YL21, a naphthoquinone derivative with two dithiocarbamate groups, forms stable Cu–thiolate complexes that efficiently deliver Cu to mitochondria [97]. Like DSF or more likely its metabolite DDC, YL21 significantly increases intracellular Cu levels when combined with Cu^2+^, leading to mitochondrial dysfunction and protein aggregation. However, YL21 is more soluble in aqueous environments than DSF, which can improve Cu retention and reduce the need for additional formulation strategies to maintain bioavailability. In contrast, rhenium(I) complexes like Re5 do not bind Cu directly but instead act as indirect Cu carriers [98]. These complexes coordinate with nitrogen-donor ligands, such as bipyridine or phenanthroline, which do not strongly chelate Cu but can indirectly promote Cu accumulation. Once inside the mitochondria, Re5 can facilitate the reduction of Cu^2+^ to Cu^+^ in the presence of cellular reductants like NADH and GSH, producing highly reactive hydroxyl radicals through Fenton-like reactions. This approach bypasses the solubility and stability issues faced by direct Cu chelators like DDC, allowing for more precise control over intracellular Cu distribution and oxidative stress. Additionally, it is believed that the positive charge of rhenium complexes helps drive them into the negatively charged mitochondrial matrix, where the buildup of reduced Cu can increase oxidative stress and mitochondrial damage [98].

### 3.2. Cu Chelators

Contrary to the potential of Cu ionophores to augment antitumor responses, Cu chelation has been explored as a way of reducing intracellular Cu levels and inhibiting Cu-driven cancerous cell growth (i.e., cuproplasia) [99,100]. This raises an important dilemma: is Cu beneficial or detrimental in the context of cancer therapeutics? Commonly used chelators include D-penicillamine, trientine, and tetrathiomobdylate (TM) (Table 2), which differ from ionophores in that the binding coefficient to Cu is generally stronger and leads to the sequestration of Cu instead of transport of Cu as would be driven by ionophores [101]. Cu chelators target cell metabolism, kinase pathways, and immunomodulating pathways, including NF-κB, the inhibition of which potentially leads to a reduction of various cytokines, including IL-1b, IL-6, and IL-8, all of which are known to modify immune components of the tumor microenvironment (TME). Cu chelation has shown efficacy in combination with monoclonal antibody therapy [102], immune activation [103], and oncolytic virotherapy [104]; however, there are few studies on the direct role of Cu chelation in antitumor immune response, and more research is needed.

**Table 2:** Commonly used Cu chelators and ionophores.

Cu-like Ionophores	Cellular Effect	Immune Effect
Disulfiram [11,14,105,106,107]	* ↓NF-κB, PSM, ALDH, * ↑ROS	* ↑ICD, PD-L1; ↓RA
Clioquinol [108]	* ↓NF-κB, PSM	n.d.
Pyrrolidine dithiocarbamate [108,109,110]	* ↓NF-κB, PSM, ↑CSP3	↓TNF-α, ↓IL-12, ↑IL-10
Elesclomol [111]	↑ROS, ↓ATP7A	n.d.
**Cu Chelators**		
Penicillamine [112,113,114]	↑ROS, ↑dsDNA breaks	↓TC, BC, NK, NP
Tetrathiomobdylate [115,116]	↓NF-κB, RAS/MAPK	↓TNF, IL-2, IL-4, IL-5, IFN-γ; ↑CD4+ infiltrate
Trientine [117]	↓Angiogenesis	↓IL-8
Curcumin [118,119]	↓NF-κB, ↑ROS	↓IL-1, IL-6, IL-8, IL-12

Cu-like ionophores are listed separately from copper chelators based on their predominant mechanism of action. Effects marked with an asterisk (*) were observed only under conditions of exogenously added Cu, whereas unmarked effects were reported in the absence of Cu supplementation. Arrows indicate the direction of change relative to control conditions. PSM, proteasome; ICD, immunogenic cell death; RA, retinoic acid; CSP3, caspase 3; TC, T cells; BC, B cells; NK, natural killer cells; NP, neutrophils, n.d. = no data.

## 4. Cu’s Role in the Immune System

**Figure 3:**
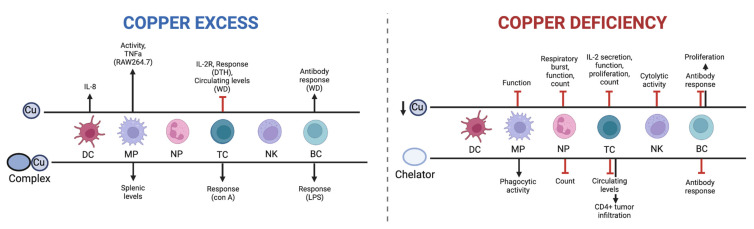
Effect of Cu excess or Cu deprivation on immune cells. Supraphysiological Cu generally leads to an activation of processes promoting immune response, while Cu deficiency leads to an inhibition of immune-activating pathways. Cu excess includes Cu ions or Cu in complexation, and Cu deficiency includes dietary Cu deficiency or Cu chelation. DC = dendritic cell, MP = macrophage, NP = neutrophil, TC = T cell, NK = natural killer cell, BC = B cell, DTH = delayed-type hypersensitivity, WD = Wilson’s disease, con A = Concanavalin A, LPS = lipopolysaccharide. Created with Biorender. Heroux, D. (2026) https://BioRender.com/feifezk.

It is clear that Cu can modulate the immune system, but it is not clear whether Cu excess or Cu deficiency would be best. This is because there is a poor understanding of how Cu can regulate immune functions in general and in the context of cancer immunotherapy in particular. This gap is particularly important in oncology, where Cu modulation may simultaneously influence immune effector function, immune suppression, and response to immune checkpoint inhibition, as summarized in Figure 3.

### 4.1. Cu and the Immune System

Cu levels above physiological requirements appear to have varied effects on the innate and adaptive immune system. The studies summarized below are largely derived from non-tumor contexts but are included here to define Cu-dependent immune phenotypes that may influence tumor immunity and immunotherapy outcomes. White et al. found that treating macrophages with Cu led to enhanced activity and intracellular killing of *E. coli* [120]. This effect was likely due to the Cu-catalyzed production of the hydroxyl radical from H_2_O_2_, allowing physiological levels of H_2_O_2_ to be lethal to bacteria [121]. An examination of the response of metallic allergens on dendritic cells (DCs) found that CuSO_4_ caused the release of IL-8 [122], a marker of DC activation. A study in mice fed with high levels of Cu exhibited a significant decrease in delayed-type hypersensitivity responses (DTH) associated with T cell recruitment [123]. Further, a study assessing long-term high Cu intake in young men suggested that the Cu-containing diet decreased the levels of IL-2 receptor, which was associated with regulating T cell proliferation [124]. These results are in agreement with observational studies of patients with Wilson’s disease (WD), a genetic disease caused by a mutation in the ATP7B gene. A study by Czlonkowska et al. determined the effect of WD on immune function and found that cell-mediated immunity was impaired, yet antibody response was greatly increased [125].

As noted already, Cu complexes are being considered as therapeutics, and these complexes have been shown to have a stimulatory effect on the immune system in mice. For example, a study found that injection of Cu_2_(3,5-diisopropylsalicylate)_4_ caused splenomegaly with increased splenic macrophage levels and enhanced T cell and B cell response [126]. It is not clear whether the effects were due to Cu or the Cu complex. As noted above, some Cu complexes actually can act like Cu ionophores, bringing Cu into cells. The effect of Cu is, however, uncertain as there are not many studies highlighting the impact of Cu deficiencies on immune function. Studies have suggested that Cu deficiencies can result in decreased cell-mediated and humoral immunity [127] and increased rates of infection and mortality in animals maintained on Cu-deficient diets [128]. Impaired cellular function may be a result of reduced Cu-dependent enzyme activity, and the effects of dietary and chelation-induced Cu deficiency are summarized below. In cancer, these immune shifts are expected to affect antigen presentation, cytotoxic effector function, and myeloid polarization within the tumor microenvironment, motivating the mechanistic and therapeutic sections that follow. Much of the evidence linking Cu modulation to immune activation or suppression is derived from preclinical and rodent models, underscoring the need for caution when extrapolating these findings to human cancer immunotherapy.

### 4.2. Dietary Cu Deficiency

The effects of insufficient dietary Cu on neutrophils have long been recognized, causing a decrease in circulating neutrophils in animals and humans. Low levels of Cu in the diet can be associated with the inhibition of respiratory burst and microbicidal functions [129]. Babu & Failla demonstrated that cellular Cu status, respiratory burst, and yeast-killing ability of peritoneal macrophages decrease in severely Cu-deficient rats [130]. Further, in a study comparing spleens from Cu-adequate and Cu-deficient rats, the NK cells from the Cu-adequate rats were five to sevenfold more cytotoxic [131]. Cu deficiency causes a general reduction in T lymphocytes, predominantly CD4+ cells, while the decrease in the CD8+ cells is less pronounced [132]. The B cell response required for antigen processing and T cell coordination, as well as antibody production, is seemingly impaired in Cu-deficient rats [133]. This does not appear to be due to a reduction in B cells. Some studies have actually suggested an increase in B cell populations when there is Cu deficiency [127]. Finally, morphological studies in mice and rats have shown that Cu deficiency is characterized by small thymuses and enlarged spleens, accompanied by a notable decrease in IL-2 secretion by rodent splenocytes. IL-2 production is required for T cell proliferation. The referenced studies in this section assessed how reducing Cu levels in the diet may affect immune cells, but more studies have actively pursued chelation therapy to engender Cu reductions. In oncology, these findings suggest that systemic Cu depletion has the potential to dampen antitumor immunity by reducing NK and T cell function, which is directly relevant when Cu chelation is considered alongside immunotherapy.

### 4.3. Cu Chelation

**Figure 4:**
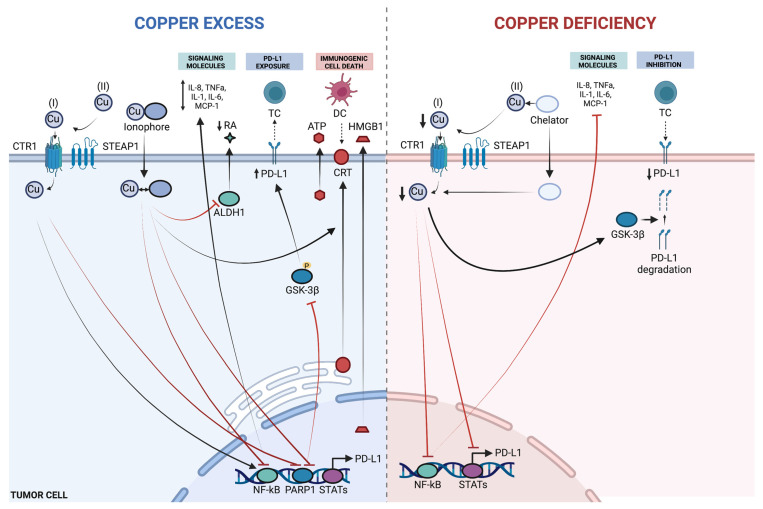
Potential effects of Cu excess or Cu deprivation in tumor cells. Supraphysiological Cu leads to modulation of cytokine production, PD-L1 expression, and activation of immunogenic cell death, while Cu deficiency inhibits cytokine expression while decreasing PD-L1 exposure. Excess Cu includes Cu ions or Cu in complexation, and Cu deficiency includes dietary Cu deficiency or Cu chelation. DC = dendritic cell, TC = T cell, RA = retinoic acid, Cu(I) = Cu^+^, Cu(II) = Cu^2+^, ATP = adenosine triphosphate, CRT = calreticulin. Solid arrows indicate proposed activating or promoting effects, dashed arrows indicate regulatory or indirect effects, and red lines denote inhibitory interactions. Created with Biorender. Heroux, D. (2026) https://BioRender.com/w6uuz96.

The effects on immune cells achieved by Cu chelation methods are similar to what has been observed when evaluating dietary Cu restrictions, but there are notable exceptions. Interestingly, Cu depletion through tetrathiomolybdate caused an increase in CD4+ T cell tumor infiltration in a murine breast cancer model, while decreasing myeloid-derived suppressor cell (MDSC) levels [115]. The general decrease in the number and/or function of neutrophils, T cells, B cells, and NK cells was comparable to that observed when Cu deficit diets were provided [113]. This illustrates that the immune consequences of chelation can be context- and model-dependent, emphasizing the need to interpret systemic immune suppression versus intratumoral immune remodeling separately. Taken together, these findings suggest that both Cu excess and Cu deprivation can remodel tumor cell signaling and immune interactions in distinct ways, influencing cytokine production, immune checkpoint regulation, and stress-associated death pathways (Figure 4).

### 4.4. Cu and Immunogenic Cell Death of Cancer Cells

Regulated cell death (RCD) is a type of cellular demise that relies on dedicated molecular machinery, in contrast to the instantaneous demise of cells exposed to physical, chemical, or mechanical forces [134]. Initially proposed to be immunologically silent [130], researchers have shown that various forms of RCD can be immunogenic, including chemotherapy and radiotherapy-induced apoptosis that was observed to activate an antitumor adaptive immune response [135]. In 1953, Mole described a regression of tumors outside of the irradiated region, using the term abscopal, meaning “away from the target” in Latin [136]. The abscopal effect was questioned for several decades due to its rarity of occurrence [137], while in 2005, Casares et al. found that doxorubicin induces a caspase-dependent immune response [135]. This form of RCD has been referred to as immunogenic cell death (ICD) and was defined in 2018 by the Nomenclature Committee on Cell Death as ‘a form of RCD that is sufficient to activate an adaptive immune response in immunocompetent syngeneic hosts’ [134]. This requires two essential elements, including activation of cytotoxic T lymphocyte (CTL)-driven adaptive immunity as well as the generation of immunological memory [138].

The immunogenicity of cell death depends on various factors, including the intrinsic antigenicity of the cells, as well as the presence of adjuvant signals. ICD involves spatiotemporal exposure or release of danger signals, which are required for the recruitment of antigen-presenting cells (APCs). These signals are collectively referred to as damage-associated molecular patterns (DAMPs) [139], and include the exposure of calreticulin (CRT) on the cellular surface, the secretion of high mobility group box 1 (HMGB1), and the release of adenosine triphosphate (ATP) [138,140,141]. These signals bind pattern recognition receptors (PRRs) in DCs and subsequently recruit CTLs into the tumor microenvironment [142,143].

Anticancer therapies, including chemotherapy, targeted therapy, and radiation therapy, have been shown to elicit clinically relevant ICD responses [144,145,146], and there is evidence that the combination of certain ICD inducers with immune checkpoint inhibitors (ICIs) leads to improved efficacy [147,148,149,150,151]. Despite clinical potential, only a few bona fide ICD inducers have been employed for use in combination therapy with ICIs [144,146,152], although numerous FDA-approved ICD inducers are being investigated for this purpose [153,154,155,156,157,158]. This framework is relevant to Cu biology because intracellular Cu delivery can trigger ER stress and redox disruption [159], mechanisms that overlap with established ICD pathways and can be leveraged in combination with immune checkpoint blockade.

### 4.5. Approved ICD-Inducing Treatments with an Emphasis on Disulfiram

Considerable efforts have been made in using combination therapy of ICD-inducing agents and ICIs in colon and rectal cancers, with the focus largely on oxaliplatin combined with PD-1/PD-L1 blocking antibodies [160]. Multiple trials have indicated that oxaliplatin is a more favorable ICI combination agent than cisplatin [161,162], and this is believed to be due to oxaliplatin’s potent role as an ICD inducer [163]. Supporting this is the finding that single-nucleotide polymorphisms (SNPs) in ICD-related genes could affect clinical outcomes in patients treated with oxaliplatin [164]. Recently, a combination of the antibody–drug conjugate enfortumab vedotin (EV) and pembrolizumab was approved for patients with locally advanced or metastatic urothelial cancer [150]. EV is a notable inducer of ICD, causing ER stress and immune cell recruitment, which contribute to its clinical efficacy in combination with PD-1 therapy [150].

Disulfiram (DSF), a long-approved drug for the treatment of alcohol use disorder, has gained attention for its potential anticancer properties. In biological systems, DSF is rapidly reduced to diethyldithiocarbamate (DDC), which readily binds Cu to form Cu(DDC)_2_. This metabolite, rather than DSF itself, is believed to mediate most of the anticancer effects associated with DSF [165,166]. While DSF alone generally requires low micromolar concentrations to elicit anticancer effects, Cu complexation substantially increases potency, with ICD induction reported at nanomolar levels in preclinical models [92,166]. Cu(DDC)_2_ has been shown to augment proteasomal degradation through inhibition of p97/NPL4, disrupt redox balance, and induce apoptosis and ICD signaling [106,165,166,167]. A schematic of this metabolic conversion and Cu complex formation is shown in Figure 2.

Recent studies have demonstrated that DSF/Cu can activate ICD markers in multiple tumor types. In human colorectal cancer (CRC) models, DSF/Cu treatment led to the cell surface exposure of CRT and HSP70, which promote the phagocytosis of tumor cells by APCs and are associated with the development of ICD [138,140]. A corresponding xenograft study confirmed in vivo induction of these markers, providing early evidence for the potential of ionophore-delivered Cu as an ICD inducer [10,146,148].

Beyond CRC, DSF/Cu has also shown promise in other malignancies where ICD and immune modulation are relevant, including breast cancer (BC). Although BC was not among the initial cancer types studied clinically with ICIs, there have been recent approvals for the indication of ICIs in triple-negative breast cancer (TNBC), and numerous ongoing trials for TNBC and other subtypes, including HER2-positive cancer [168,169,170,171,172]. There is evidence that cancer stem cells (CSCs), believed to be responsible for tumor progression and metastasis [173], also interact with various immune cells, promoting immune silencing and avoidance of destruction [174]. Irradiation therapy (IR) is a standard treatment for BC and has been reported to induce ICD in breast and other cancers, enhancing the efficacy of ICIs [175,176]. However, consistent with evidence that breast cancer stem cells (BCSCs) are treatment-resistant, Sun and colleagues demonstrated that IR triggered reduced levels of ICD signals in BCSCs [12]. A common molecular target for CSC inhibition is ALDH, which is believed to cause resistance by removing genotoxic aldehydes in BCSC [173]. Because DSF is a pan-ALDH inhibitor, its combination with Cu has been explored as a strategy to target CSCs and sensitize them to ICD-inducing therapies [177]. Sun et al. found that resistance in ICD induction of irradiated cells was removed through pre-treatment with DSF/Cu by assessing ICD markers of CRT, HSP90, and HMGB1 [12], representing the first study to use disulfiram with Cu to induce ICD in CSCs.

While several recent approvals of ICIs for hepatocellular carcinoma (HCC) have significantly improved management of the disease [178], most patients yield limited benefits from immune-based therapies [179]. The immunosuppressive microenvironment in the liver is influenced by Kupffer cells, MDSCs, Tregs, and anti-inflammatory cytokines, and a plausible strategy in enhancing immunotherapy response is the conversion to an inflammatory TME [180]. DSF has been used to inhibit HCC tumor-initiating cells (TICs) [181], and a recent study by Gao and colleagues demonstrated the potential of DSF/Cu to cause activation of several ICD markers, including CRT, HMGB1, ATP, and IFN [11]. Significantly, a cancer vaccination model was used to provide the first in vivo ICD validation of DSF/Cu, and synergistic antitumor activity was demonstrated in combination with CD47 blockade. DSF/Cu-treated cells underwent a significant gene enrichment in Cu response, suggesting the potential of ionophore-mediated Cu delivery for further applications of ICD induction. Despite DSF being the most frequently delivered form, future studies are expected to clarify the clinical potential of DDC in complexation with Cu.

## 5. Cu, Cu Complexes, and PD-L1

Other efforts to induce ICD with Cu have involved the synthesis of Cu(II) complexes to generate intracellular ROS. Kaur and colleagues prepared several compounds containing a Schiff base ligand (known to generate ROS in combination with Cu) with various lipophilic polypyridyl ligands (known to facilitate localization in the ER) [182]. The lead diphenyl-1,10-phenanthroline-bearing complex was cytotoxic to both bulk breast cancer cells and BCSCs at sub-micromolar concentrations, induced various DAMPs, including CRT and ATP, and promoted BCSC phagocytosis by macrophages. This represented the first cytotoxic Cu(II) complex to induce ICD in BCSC, and provided early rationale for the use of Cu in ICD. However, when considering the use of Cu and ICD induction, it is our contention that these should be considered in the context of ICIs such as the antibodies that have been developed to target Programmed Cell Death Ligand (PD-L1).

PD-L1 is a transmembrane protein normally expressed by DCs, MPs, some activated T cells and B cells, and tumor cells, in which it acts in a mechanism to escape antitumor immune responses [183]. The PD-1/PD-L1 pathway maintains immune tolerance in the tumor microenvironment, controlling T cell activation and cytokine secretion, leading to PD-1-mediated T cell exhaustion and reduced cytotoxicity against tumor cells [183]. This negative interaction can be inhibited by anti-PD-1/anti-PD-L1 antibodies, and since the approval of pembrolizumab for the treatment of advanced melanoma in 2014, PD-1/PD-L1 ICIs have been approved for use in many other tumors [184], although their efficacy is limited by various mechanisms of resistance in certain patients and side effects in others [185]. PD-L1 protein expression on tumor cells was the first potential predictive biomarker for sensitivity to ICIs, and currently remains the best validated marker for cancer immunotherapy [186,187]. While high PD-L1 levels have been associated with increased tumor immune infiltration [188], its expression is correlated with worse prognosis in many tumors [189,190]. In the context of this review, it is important to consider the role of Cu and PD-L1 expression and targeted therapy.

Various mechanisms may lead to increased PD-L1 expression, including tumor microenvironment (TME) release of pro-inflammatory cytokines such as IFN-γ, TNF-α, and IL-6, which activate signaling pathways including EGFR, PI3K, and AKT [191]. Cu(II) ions have been found to activate EGFR in the absence of its ligand and cause activation of the AKT and ERK pathways [192], and recently, Voli and colleagues reported that intra-tumor Cu influences PD-L1 levels [13]. The addition of Cu was shown to increase PD-L1 mRNA and protein levels, and upregulated the same target gene set as IFN-γ, while Cu chelators decreased PD-L1 expression by inhibiting cancer cells’ response to IFN-γ, TNF-α, and IFN-α/β. Cu chelation inhibited EGFR phosphorylation, causing an accumulation of ubiquitinated PD-L1 and subsequent proteasomal degradation, and decreased STAT3 phosphorylation, reducing the expression of several STAT target genes. Decreasing Cu levels in immune-competent tumor-bearing mice led to an increase in tumor-infiltrating CD8+ T cells and NK cells, demonstrating the therapeutic potential of Cu chelation in the context of decreased PD-L1 expression levels. With this in mind, one may question the use of Cu-like ionophores that could mediate increased levels of Cu in tumor cells. However, this needs to be considered in the context of combinations of Cu-like ionophores with PD-L1 targeted therapeutics.

Due to the formation of the highly potent anticancer complex Cu(DDC)_2_ when DSF is administered with Cu, DSF is most frequently studied in combination with Cu in cancer research [91]. However, as many tumors contain elevated levels of Cu, this may be sufficient for the formation of active levels of Cu(DDC)_2_ [193], and due to other less studied mechanisms, there has been some clinical interest in DSF given without Cu [194,195]. In a recent preclinical study, Zheng and colleagues uncovered a link between DSF treatment-mediated overexpression of IRF7, a regulator of type I IFN, and upregulation of PD-L1 [15]. IRF7 binds to the PD-L1 promoter, causing overexpression, a function that is attenuated by methyltransferases such as DNMT1. DNMT1 inhibitors such as decitabine have been studied clinically to improve the response of anti-PD-1/PD-L1 inhibition [196,197]. While DNMT1-mediated PD-L1 upregulation promotes immune escape, combination treatment with ICIs promotes an antitumor immune response. Such data provides a rationale to combine PD-L1 inhibitors with Cu-like ionophores such as DDC. Interestingly, DSF treatment was found to inhibit DNMT1, and while this resulted in no antitumor effects as a single agent, the treatment decreased intratumoral T cell infiltration. Surprisingly, the combination with anti-PD-1 mAb led to a synergistic antitumor immune response, representing a novel therapeutic strategy for metastatic triple-negative breast cancer (TNBC) and other tumors. Another recent study also showed synergy between DSF and anti-PD-1 treatment in melanoma, uncovering a stimulatory effect of DSF on CD8+ T cells [198]. The authors of this study also noted that DSF treatment directly bound LCK, the first molecule to be recruited to the TCR complex. This enhanced its kinase activity and increased T cell effector response and antitumor immunity. The role of basal Cu in binding the DDC metabolite of DSF in this study is unclear, and further studies are needed to understand this and the necessity of exogenous Cu addition for DSF-mediated immune activation, knowing that DSF is rapidly metabolized to DDC.

As noted above, disulfiram and or DDC inhibit aldehyde dehydrogenase (ALDH), and this is why it is used to prevent alcohol consumption. ALDH is an enzyme responsible for oxidizing aldehydes resulting from metabolic processes, and is also important for the maintenance and differentiation of stem cells [199]. ALDHs regulate various pathways in cancer to promote tumorigenesis and cancer stem cell signaling, including minimizing ROS production and enhancing retinoic acid (RA) signaling [200,201]. ALDH is a negative prognostic marker for most tumor types [202,203] and promotes an immune suppressive landscape by inducing Treg and modulating macrophage polarization through the production of RA by multiple cell types [200,204]. ALDH is positively correlated with PD-L1 levels in CRC and lung cancer patients [205,206], and PD-L1 is known to promote various factors that maintain CSC stemness, including OCT4 and Nanog through activation of the PI3K/AKT pathway [207]. Thus, dual ALDH and PD-1/PD-L1 axis inhibition is a reasonable anticancer strategy.

Intratumoral Cu level has been found to have an inverse correlation between ALDH protein levels in osteosarcoma (OS) cells, with ALDH^high^ highly metastatic cell lines having low Cu levels and an ALDH^low^ low metastatic line having high levels of Cu [208]. Many studies have linked DSF(DDC)/Cu to ALDH inhibition [18,209], with evidence suggesting that DSF(DDC)/Cu targets a stem-cell-like ALDH population and reports antitumor activity against an ALDH^high^ population in vivo [210]. In contrast to this, Skrott et al. recently suggested that anticancer activity of DSF(DDC)/Cu is not due to ALDH inhibition, as neither DSF nor DSF/Cu directly inhibits ALDH in a short-term assay. They argued that the long DSF(DDC)/Cu exposure times used in previous studies caused cell permeability, confounding results from the ALDEFLUOR assay used to measure ALDH function. They also argued that the bona fide inhibitor of ALDH was the non-toxic DSF metabolite S-methyl-N,N-diethylthiocarbamate-sulfoxide (Me-DTC-SO) [211], which is only generated in vivo and the formation of which is minimized when DSF(DDC) is in the presence of Cu. Specifically, when Cu(DDC)_2_ is formed, it can be an active anticancer agent targeting NPL4, a subunit of the p97/VCP segregase [166]. Altogether, this provides compelling evidence that challenges the long-held belief that DSF(DDC)/Cu targets ALDH inhibition as a major anticancer mechanism.

While the precise role of DSF(DDC)/Cu in ALDH inhibition is under debate, many recently developed ALDH inhibitors have shown promising efficacy in tumors [212], and a recent in silico screen of natural products for inhibitors of ALDH isoforms led to the synthesis of a rutin–Cu complex that had sub-micromolar activity in a breast cancer cell line. Although there is promising evidence for the role of Cu and Cu compounds in immunomodulatory ALDH inhibition, contradictory reports and limited evidence indicate that further research is needed.

It is also worth noting that Cu has been shown to initiate an inflammatory state in rats, causing the production of reactive oxygen species (ROS) and the activation of a downstream target of nuclear factor kappa-light-chain-enhancer of activated B cells (NF-κB) [16] as well as activate NF-κB and TNF-α in the spleen and thymus of chickens [213,214]. Cu also caused the overexpression of NF-κB-target cytokines in mice when given in feed [215], and Cu chelation through tetrathiomolybdate (TM) led to a decrease in NF-κB-mediated pro-inflammatory cytokines in microglial cells in mice [216] while inhibiting NF-κB in breast cancer cells [217]. Conversely, Kanemaru et al. found that Cu, when given as Cu(II) ions or in a peptide complex, inhibited NF-κB in ovarian cancer cells and Jurkat T cells, and a number of other Cu complexes have demonstrated NF-κB pathway inhibition in cancer [218,219,220]. NF-κB is a transcription-factor family comprising five subunits that controls expression of target genes including IL6, TNF-α, BCL2, and VEGF, influencing tumor cell proliferation, inflammation, and adaptive immunity [221]. NF-κB is viewed as a critical link between inflammation and tumorigenesis, and in certain tumor environments, it can promote tumor proliferating effects through inflammation or immunosuppression [222]. Tumor cells produce a variety of NF-κB-induced cytokines and chemokines that influence the recruitment and activation of immune cells, and NF-κB mediates transcription and protein stability of PD-L1 in tumor cells, contributing to CD8+ T cell exhaustion [223].

Although there is evidence that NF-κB activation and signaling in tumor cells may increase patient response to ICIs [224,225], NF-κB inhibition can also be beneficial for patient response. NF-κB inhibition downregulates genes involved in metastasis and angiogenesis in tumor cells [226] and has numerous effects on immune cells in the TME, including stimulation of DCs, T cells, and NK cells, and relieving suppression exerted by MDSCs and Tregs [223]. A well-studied compound is bortezomib, which inhibits NF-κB as a by-product of its proteasome inhibition function [227] and has been shown to have synergistic effects with anti-PD-1 therapy [228], with ongoing clinical interest [229].

While pyrrolidine dithiocarbamate, 8-hydroxyquinoline metabolites, and other Cu-complexing compounds have been shown to inhibit NF-κB in cancer [230,231], DSF(DDC) is the most studied [105,232,233] and, as an inhibitor of the proteasome system, DSF/Cu inhibits degradation of inhibitor-kB (IkB), leading to suppression of NF-κB nuclear translocation and activation. The activity of DSF/Cu has been used to inhibit 5-fluorouricil-induced NF-κB activation [105] and the reversal of chemoresistance in colon and breast cancers [105,234]. Although there is a dearth of studies that examine DSF(DDC)/Cu’s NF-κB inhibition in cancer and the resulting modulation of immune phenotype, DSF has been examined for the inhibition of NF-κB-mediated cytokine output in a phase 2 clinical trial for coronavirus disease 2019 (COVID-19). The primary outcomes were a change in plasma inflammatory biomarker levels (e.g., IL-6 and IL-b) and viral load on days 5, 15, and 31 [235]. DSF has also been examined clinically with minor benefit in HIV latency reversion [236], and due to the context-dependent, possibly contradictory findings from studies of Cu and NF-κB interactions, further studies are needed to understand the role of DSF(DDC) in antitumor and anti-viral immunity for the potential of this diversely active compound to be realized.

While Cu modulation has clear potential to influence cancer progression and treatment response, particularly in the context of immunotherapy, its therapeutic development was historically limited by concerns around toxicity and formulation challenges. In oncology, however, toxicity is often acceptable within a defined therapeutic index, and Cu’s redox activity may offer advantages in promoting tumor-selective stress and immune activation. One long-standing hurdle has been the tendency of certain Cu complexes to form insoluble precipitates, limiting their viability as drug candidates. More recently, a growing number of groups, including ours, have addressed this through nanoformulations, which can improve apparent Cu solubility, stability, and tumor targeting. This shift has opened new directions for Cu-based therapeutic design.

## 6. Cu-Based Nanomedicines

Cu-based nanomedicines have undergone a rapid evolution, transitioning from antimicrobial materials to highly engineered platforms designed to deliver immunomodulatory and cytotoxic effects. Early studies on Cu nanoparticles (Cu-NPs) and Cu oxide nanoparticles (CuO-NPs) demonstrated strong bactericidal and virucidal activity [218,219], which later led to their investigation in cancer contexts. However, their translation was hindered by dose-limiting toxicities, pro-inflammatory off-target effects, and limited capacity for tumor-specific delivery [220,237,238]. The reactive nature of Cu ions in circulation, including their interaction with serum proteins and redox cycling in non-target tissues, presented considerable challenges to systemic application [159]. Initial work with CuO-NPs in tumor models demonstrated dose-dependent tumor suppression, but also substantial collateral tissue damage [239], reinforcing the need for more sophisticated delivery strategies that could direct Cu to tumors while minimizing systemic exposure.

To overcome these issues, second-generation Cu nanomedicines incorporated Cu(II) ions into stabilized drug delivery systems. Two widely cited clinical examples are Vyxeos^®^ (CPX-351) and Irinophore C™, which employ metal-coordinated drug encapsulation. Vyxeos^®^ uses Cu-bound daunorubicin co-encapsulated with cytarabine at a synergistic 5:1 molar ratio, with Cu stabilizing the complex and modulating drug-release kinetics such that the drug-to-drug ratio is maintained after IV administration [240,241,242]. Irinophore C™ (which did not advance to the clinic) demonstrated that intraliposomal Cu could interact with irinotecan’s lactone and quinoline groups, significantly prolonging drug retention compared with liposomes loaded using pH gradients alone or when other metals were used [243]. In this formulation, Cu confers unique properties. Comparative studies showed that substitution with other divalent cations such as Zn^2+^, Mg^2+^, or Mn^2+^ failed to achieve similar retention [244,245,246,247]. Notably, Cu’s ability to coordinate both the drug and the inner phospholipid leaflet was proposed to reduce passive permeability, further enhancing retention. Although this irinotecan formulation did not progress to clinical trials, in part due to the approval of Onivyde, the broader concept of using metals to coordinate drug molecules was pursued further. Together, these early studies established Cu coordination as a viable strategy for modulating drug retention and pharmacokinetics, providing a foundation for subsequent nanomedicine technologies.

Building on these early studies, our group developed a formulation strategy called Metaplex, which uses Cu coordination within liposomal carriers. Cu(II) solutions are first encapsulated into clinically relevant DSPC/cholesterol liposomes in the ~100 nm size range, then mixed with metal-binding ligands such as DDC (the disulfiram metabolite) and clioquinol to form Cu complexes inside the aqueous core [92,248]. This approach allows poorly soluble ligands to be incorporated as stable Cu complexes. Injectable Cu(DDC)_2_ liposomes prepared using this method were shown to reduce tumor growth in preclinical models [93], and the same strategy was later applied to Cu(CQ)_2_‚ demonstrating compatibility with a range of structurally diverse ligands [249]. Importantly, liposomal encapsulation of Cu complexes was associated with improved tolerability and model-dependent modulation of intratumoral stress and immune-related signaling in preclinical colorectal cancer models, with divergent responses observed between CT26 and MC38 tumors following short- and long-term Cu exposure, underscoring the importance of formulation-controlled Cu delivery [250,251].

Other Cu-based nanomedicine strategies have focused on developing nanoparticles that release Cu along with complementary agents to induce cancer cell death. One example is a core–shell nanoparticle (CuP/Er) that releases Cu(II) and erastin in the acidic tumor microenvironment [252]. Erastin blocks a key antioxidant transporter, leading to glutathione depletion and increased lipid peroxidation, while Cu(II) binds to lipoylated TCA cycle proteins and disrupts mitochondrial function, promoting cuproptosis. In MC38 and 4T1 tumor models, this combination induced ICD characterized by calreticulin exposure, HMGB1 release, and ATP secretion, and significantly inhibited tumor growth when combined with anti-PD-L1 antibodies. Another approach integrates the IDO1 inhibitor NLG919 to overcome the immunosuppressive tumor microenvironment [253]. This strategy uses a biomimetic nanoparticle (ECNM) combining Cu^2+^, elesclomol (ES), and NLG919 for enhanced tumor targeting and stability. The NLG919 component blocks IDO1, reversing immune suppression and promoting DC maturation and T cell activation, while the Cu^2+^ and ES components induce cuproptosis. In 4T1 tumor models, this combination induced hallmarks of ICD and contributed to durable antitumor responses.

Building on the concept, some approaches considered using live immune cells as carriers. MetaCells, for example, are cellular Trojan horses that incorporate Fe-Cu metal–organic frameworks (MOFs) into live neutrophils, taking advantage of the natural tumor-homing capability of these cells [254]. Neutrophils are drawn to inflamed tumor sites, allowing targeted delivery of Fe-Cu MOFs directly into the tumor microenvironment. Once released, these MOFs generate reactive oxygen species (ROS), deplete glutathione, and activate both cuproptosis and ferroptosis, promoting antigen presentation and T cell activation. This strategy produced robust antitumor effects in 4T1 models, including near-complete tumor regression.

MOFs, like those used in the MetaCell platform, are highly porous, crystalline structures that allow precise control over the release of Cu^2+^ and other therapeutic agents within the tumor microenvironment. These frameworks can be engineered to respond to specific triggers, enhancing the selectivity and potency of Cu-based therapies. For example, a CaO_2_@Cu-MOF nanoreactor was designed to release Cu^2+^ and the BRD4 inhibitor JQ-1 in response to GSH-rich and acidic conditions, generating Cu^+^, blocking ATP7B, and producing oxygen to reduce hypoxia, promoting antigen presentation and T cell activation in CT26 colorectal cancer models [255]. Luo et al. developed ES-Cu-MOF nanoparticles by incorporating elesclomol and Cu^2+^ within a Cu-based MOF, allowing for pH-responsive release in the intracellular environment [256]. In fibrosarcoma models, the released cargo led to mitochondrial stress, loss of FDX1, and features of ICD, including dendritic cell activation.

External activation strategies, including photodynamic therapy (PDT) and sonodynamic therapy (SDT), have also been explored to improve the performance of Cu nanomaterials. PDT uses light to activate Cu-containing photosensitizers, generating reactive oxygen species (ROS) through energy transfer processes. However, the hypoxic tumor microenvironment (TME) can limit oxygen availability, reducing ROS production. To address this, systems like Au@SiO_2_@Cu_2_O nanocomposites have been developed, incorporating an oxygen-enriched core to boost ^1^O_2_ production under light activation [257]. These designs also take advantage of Cu’s ability to deplete intracellular GSH, lowering the antioxidant capacity of tumor cells and amplifying oxidative damage [257,258,259]. SDT, in contrast, uses ultrasound to penetrate deeper tissues and activate Cu nanostructures through mechanical and thermal effects. For example, Yan et al. developed Cu_2_O nanocubes coated with graphene quantum dots (GQDs) to create a sonosensitizer that releases Cu^+^ ions in acidic conditions while promoting ROS production under ultrasound [260]. The GQD shell stabilizes the Cu_2_O core and improves electron transfer under ultrasound, enhancing ROS formation without requiring high oxygen levels. Similarly, Cu-cysteamine nanoparticles have been shown to generate ROS upon ultrasound activation, demonstrating efficacy in preclinical tumor models [261].

## 7. Conclusions

This review highlights the growing interest in Cu as a therapeutic target in cancer, particularly in the context of immune modulation. Accumulating evidence indicates that altering Cu availability can influence tumor growth, immune activation, and response to therapy, while advances in nanotechnology are enabling more controlled and selective delivery of Cu-based agents. Together, these findings support a promising, though still incompletely understood, role for Cu in cancer treatment.

For Cu-based therapies to advance clinically, safety considerations remain critical. High doses of orally administered Cu^2+^ are associated with gastrointestinal and hepatic toxicity; however, preclinical studies using Cu-based Metaplex formulations and Cu-containing liposome controls have not shown overt toxicity at doses well below established toxic thresholds in mice [92,93,262]. As with other anticancer strategies, some degree of toxicity may be acceptable if it is predictable and manageable through dose optimization, formulation design, and scheduling. Rigorous safety evaluation will therefore be essential in future clinical development.

Metal–compound complexes, including Cu-containing formulations, are increasingly recognized as being more therapeutically effective than parent compounds alone [263,264]. These complexes can engage distinct intracellular pathways or induce unique stress responses, such as ROS generation and ER stress [265,266]. In oncology, Cu-delivering systems may offer an alternative or complement to platinum-based therapies, which are often limited by chemoresistance and systemic toxicity.

Two opposing strategies, Cu chelation and Cu delivery, have both shown potential to suppress tumor growth, but each presents distinct challenges. Chelation disrupts Cu-dependent processes critical to cancer cell metabolism but may also impair immune function. In contrast, Cu overload can promote oxidative stress and immunogenic cell death while enhancing certain immune-activating pathways. Emerging evidence suggests that combining Cu delivery with immune checkpoint inhibition may yield synergistic antitumor effects [14,15,160,252,267,268].

Despite this promise, the context-dependent nature of Cu signaling complicates its therapeutic use. Key pathways such as NF-κB and ALDH1 are modulated by Cu in complex and sometimes opposing ways, underscoring the need for further mechanistic clarification. In particular, distinctions between free Cu ions and ionophore-mediated intracellular delivery remain incompletely defined. Recent studies, including work from our group, are beginning to resolve these questions by linking Cu delivery mode to immunogenic cell death, tumor Cu metabolism, and immunotherapy response [250,251,269,270,271,272]. Continued integration of these insights with advances in nanomedicine and tumor immunology is likely to be critical for the rational development of Cu-based combination therapies.

## Data Availability

No new data were created or analyzed in this study.

## References

[1] Crans D.C., Kostenkova K. (2020). Open questions on the biological roles of first-row transition metals. Commun. Chem..

[2] Pham V.N., Chang C.J. (2023). Metalloallostery and Transition Metal Signaling: Bioinorganic Copper Chemistry Beyond Active Sites. Angew. Chem. Int. Ed..

[3] Tsang T., Davis C.I., Brady D.C. (2021). Copper biology. Curr. Biol..

[4] Turski M.L., Brady D.C., Kim H.J., Kim B.-E., Nose Y., Counter C.M., Winge D.R., Thiele D.J. (2012). A Novel Role for Copper in Ras/Mitogen-Activated Protein Kinase Signaling. Mol. Cell. Biol..

[5] Tsang T., Posimo J.M., Gudiel A.A., Cicchini M., Feldser D.M., Brady D.C. (2020). Copper is an essential regulator of the autophagic kinases ULK1/2 to drive lung adenocarcinoma. Nat. Cell Biol..

[6] Lelièvre P., Sancey L., Coll J.-L., Deniaud A., Busser B. (2020). The Multifaceted Roles of Copper in Cancer: A Trace Metal Element with Dysregulated Metabolism, but Also a Target or a Bullet for Therapy. Cancers.

[7] Cen D., Brayton D., Shahandeh B., Meyskens F.L., Farmer P.J. (2004). Disulfiram Facilitates Intracellular Cu Uptake and Induces Apoptosis in Human Melanoma Cells. J. Med. Chem..

[8] Tardito S., Bassanetti I., Bignardi C., Elviri L., Tegoni M., Mucchino C., Bussolati O., Franchi-Gazzola R., Marchiò L. (2011). Copper Binding Agents Acting as Copper Ionophores Lead to Caspase Inhibition and Paraptotic Cell Death in Human Cancer Cells. J. Am. Chem. Soc..

[9] Tsvetkov P., Coy S., Petrova B., Dreishpoon M., Verma A., Abdusamad M., Rossen J., Joesch-Cohen L., Humeidi R., Spangler R.D. (2022). Copper induces cell death by targeting lipoylated TCA cycle proteins. Science.

[10] You S.-Y., Rui W., Chen S.-T., Chen H.-C., Liu X.-W., Huang J., Chen H.-Y. (2019). Process of immunogenic cell death caused by disulfiram as the anti-colorectal cancer candidate. Biochem. Biophys. Res. Commun..

[11] Gao X., Huang H., Pan C., Mei Z., Yin S., Zhou L., Zheng S. (2022). Disulfiram/Copper Induces Immunogenic Cell Death and Enhances CD47 Blockade in Hepatocellular Carcinoma. Cancers.

[12] Sun T., Yang W., Toprani S.M., Guo W., He L., DeLeo A.B., Ferrone S., Zhang G., Wang E., Lin Z. (2020). Induction of immunogenic cell death in radiation-resistant breast cancer stem cells by repurposing anti-alcoholism drug disulfiram. Cell Commun. Signal.

[13] Voli F., Valli E., Lerra L., Kimpton K., Saletta F., Giorgi F.M., Mercatelli D., Rouaen J.R.C., Shen S., Murray J.E. (2020). Intratumoral Copper Modulates PD-L1 Expression and Influences Tumor Immune Evasion. Cancer Res..

[14] Zhou B., Guo L., Zhang B., Liu S., Zhang K., Yan J., Zhang W., Yu M., Chen Z., Xu Y. (2019). Disulfiram combined with copper induces immunosuppression via PD-L1 stabilization in hepatocellular carcinoma. Am. J. Cancer Res..

[15] Zheng X., Liu Z., Mi M., Wen Q., Wu G., Zhang L. (2021). Disulfiram Improves the Anti-PD-1 Therapy Efficacy by Regulating PD-L1 Expression via Epigenetically Reactivation of IRF7 in Triple Negative Breast Cancer. Front. Oncol..

[16] Persichini T., Percario Z., Mazzon E., Colasanti M., Cuzzocrea S., Musci G. (2006). Copper Activates the NF-κB Pathway In Vivo. Antioxid. Redox Signal..

[17] Liu T., Zhang L., Joo D., Sun S.-C. (2017). NF-κB signaling in inflammation. Signal Transduct. Target. Ther..

[18] Wang N.-N., Wang L.-H., Li Y., Fu S.-Y., Xue X., Jia L.-N., Yuan X.-Z., Wang Y.-T., Tang X., Yang J.-Y. (2018). Targeting ALDH2 with disulfiram/copper reverses the resistance of cancer cells to microtubule inhibitors. Exp. Cell Res..

[19] Pino-Lagos K., Benson M.J., Noelle R.J. (2008). Retinoic Acid in the Immune System. Ann. N. Y. Acad. Sci..

[20] O’Dell B.L. (1976). Biochemistry of copper. Med. Clin. N. Am..

[21] Apelgot S., Coppey J., Fromentin A., Guille E., Poupon M.F., Roussel A. (1986). Altered distribution of copper (64Cu) in tumor-bearing mice and rats. Anticancer. Res..

[22] Coates R.J., Weiss N.S., Daling J.R., Rettmer R.L., Warnick G.R. (1989). Cancer risk in relation to serum copper levels. Cancer Res..

[23] Gupta S.K., Shukla V.K., Vaidya M.P., Roy S.K., Gupta S. (1991). Serum trace elements and Cu/Zn ratio in breast cancer patients. J. Surg. Oncol..

[24] Haddad N., Haddad H., Al-Elwee W.M. (2010). Diagnostic values of copper, zinc and copper/zinc ratio compared to histopathological examination in patients with breast tumors. Bas J. Surg..

[25] Margalioth E.J., Schenker J.G., Chevion M. (1983). Copper and zinc levels in normal and malignant tissues. Cancer.

[26] Rizk S.L., Sky-Peck H.H. (1984). Comparison between concentrations of trace elements in normal and neoplastic human breast tissue. Cancer Res..

[27] Huang C.P., Fofana M., Chan J., Chang C.J., Howell S.B. (2014). Copper transporter 2 regulates intracellular copper and sensitivity to cisplatin. Metallomics.

[28] Marinov B., Tsachev K., Doganov N., Dzherov L., Atanasova B., Markova M. (2000). The copper concentration in the blood serum of women with ovarian tumors (a preliminary report). Akush Ginekol..

[29] Yaman M., Kaya G., Simsek M. (2007). Comparison of trace element concentrations in cancerous and noncancerous human endometrial and ovary tissues. Int. J. Gynecol. Cancer.

[30] Cheng X., Zhou Y.C., Zhou B., Huang Y.C., Wang G.Z., Zhou G.B. (2019). Systematic analysis of concentrations of 52 elements in tumor and counterpart normal tissues of patients with non-small cell lung cancer. Cancer Med..

[31] Díez M., Arroyo M., Cerdàn F.J., Muñoz M., Martin M.A., Balibrea J.L. (1989). Serum and tissue trace metal levels in lung cancer. Oncology.

[32] Jin Y., Zhang C., Xu H., Xue S., Wang Y., Hou Y., Kong Y., Xu Y. (2011). Combined effects of serum trace metals and polymorphisms of CYP1A1 or GSTM1 on non-small cell lung cancer: A hospital based case-control study in China. Cancer Epidemiol..

[33] Oyama T., Matsuno K., Kawamoto T., Mitsudomi T., Shirakusa T., Kodama Y. (1994). Efficiency of serum copper/zinc ratio for differential diagnosis of patients with and without lung cancer. Biol. Trace Elem. Res..

[34] Alfaris N., Ahmad (2011). Distribution of trace elements like calcium, copper, iron and zinc in serum samples of colon cancer—A case control study. J. King Saud. Univ. Sci..

[35] Juloski J.T., Rakic A., Ćuk V.V., Ćuk V.M., Stefanović S., Nikolić D., Janković S., Trbovich A.M., De Luka S.R. (2020). Colorectal cancer and trace elements alteration. J. Trace Elem. Med. Biol..

[36] Stepien M., Jenab M., Freisling H., Becker N.-P., Czuban M., Tjønneland A., Olsen A., Overvad K., Boutron-Ruault M.-C., Mancini F.R. (2017). Pre-diagnostic copper and zinc biomarkers and colorectal cancer risk in the European Prospective Investigation into Cancer and Nutrition cohort. Carcinogenesis.

[37] Scanni A., Licciardello L., Trovato M., Tomirotti M., Biraghi M. (1977). Serum Copper and Ceruloplasmin Levels in Patients with Neoplasias Localized in the Stomach, Large Intestine or Lung. Tumori J..

[38] Yaman M., Kaya G., Yekeler H. (2007). Distribution of trace metal concentrations in paired cancerous and non-cancerous human stomach tissues. World J. Gastroenterol..

[39] Kosova F., Cetin B., Akinci M., Aslan S., Seki A., Pirhan Y., Ari Z. (2012). Serum copper levels in benign and malignant thyroid diseases. Bratisl. Lek. Listy.

[40] Vladimir Z. (2022). Content of Copper, Iron, Iodine, Rubidium, Strontium and Zinc in Thyroid Malignant Nodules and Thyroid Tissue adjacent to Nodules. J. Clin. Diagn. Pathol..

[41] Carpentieri U., Myers J., Thorpe L., Daeschner C.W., Haggard M.E. (1986). Copper, zinc, and iron in normal and leukemic lymphocytes from children. Cancer Res..

[42] Zuo X.L., Chen J.M., Zhou X., Li X.Z., Mei G.Y. (2006). Levels of selenium, zinc, copper, and antioxidant enzyme activity in patients with leukemia. Biol. Trace Elem. Res..

[43] Baharvand M., Manifar S., Akkafan R., Mortazavi H., Sabour S. (2014). Serum levels of ferritin, copper, and zinc in patients with oral cancer. Biomed. J..

[44] Shettar S.S. (2016). Estimation of serum copper and zinc levels in patients with oral cancer. J. Evol. Med. Dent. Sci..

[45] Tiwari R., David C.M., Mahesh D.R., Sambargi U., Rashmi K.J., Benakanal P. (2016). Assessment of serum copper, iron and immune complexes in potentially malignant disorders and oral cancer. Braz. Oral. Res..

[46] Chang W.-H., Lee C.-C., Yen Y.-H., Chen H.-L. (2018). Oxidative damage in patients with benign prostatic hyperplasia and prostate cancer co-exposed to phthalates and to trace elements. Environ. Int..

[47] Saleh S.A.K., Adly H.M., Abdelkhaliq A.A., Nassir A.M. (2020). Serum Levels of Selenium, Zinc, Copper, Manganese, and Iron in Prostate Cancer Patients. Curr. Urol..

[48] Margalioth E.J., Udassin R., Cohen C., Maor J., Anteby S.O., Schenker J.G. (1987). Serum copper level in gynecologic malignancies. Am. J. Obstet. Gynecol..

[49] Moyong K., Singh Y., Singh L., Devi T., Singh W. (2012). Serum copper level in different stages of cervical cancer. JMS—J. Med. Soc..

[50] Senra Varela A., Lopez Saez J.J.B., Quintela Senra D. (1997). Serum ceruloplasmin as a diagnostic marker of cancer. Cancer Lett..

[51] Ungar-Waron H., Gluckman A., Spira E., Waron M., Trainin Z. (1978). Ceruloplasmin as a marker of neoplastic activity in rabbits bearing the VX-2 carcinoma. Cancer Res..

[52] Hanahan D., Weinberg R.A. (2000). The Hallmarks of Cancer. Cell.

[53] Hanahan D., Weinberg R.A. (2011). Hallmarks of Cancer: The Next Generation. Cell.

[54] Hanahan D. (2022). Hallmarks of Cancer: New Dimensions. Cancer Discov..

[55] Lowndes S.A., Harris A.L. (2005). The role of copper in tumour angiogenesis. J. Mammary Gland. Biol. Neoplasia.

[56] Nasulewicz A., Mazur A., Opolski A. (2004). Role of copper in tumour angiogenesis--clinical implications. J. Trace Elem. Med. Biol..

[57] Kerbel R.S. (2008). Tumor angiogenesis. N. Engl. J. Med..

[58] Finney L., Vogt S., Fukai T., Glesne D. (2009). Copper and angiogenesis: Unravelling a relationship key to cancer progression. Clin. Exp. Pharmacol. Physiol..

[59] Martin F., Linden T., Katschinski D.M., Oehme F., Flamme I., Mukhopadhyay C.K., Eckhardt K., Tröger J., Barth S., Camenisch G. (2005). Copper-dependent activation of hypoxia-inducible factor (HIF)-1: Implications for ceruloplasmin regulation. Blood.

[60] Xie H., Kang Y.J. (2009). Role of copper in angiogenesis and its medicinal implications. Curr. Med. Chem..

[61] Cai L., Li X.K., Song Y., Cherian M.G. (2005). Essentiality, toxicology and chelation therapy of zinc and copper. Curr. Med. Chem..

[62] Camphausen K., Sproull M., Tantama S., Venditto V., Sankineni S., Scott T., Brechbiel M.W. (2004). Evaluation of chelating agents as anti-angiogenic therapy through copper chelation. Bioorg Med. Chem..

[63] Heuberger D.M., Harankhedkar S., Morgan T., Wolint P., Calcagni M., Lai B., Fahrni C.J., Buschmann J. (2019). High-affinity Cu(I) chelator PSP-2 as potential anti-angiogenic agent. Sci. Rep..

[64] Chan A., Wong F., Arumanayagam M. (1993). Serum ultrafiltrable copper, total copper and caeruloplasmin concentrations in gynaecological carcinomas. Ann. Clin. Biochem..

[65] Salvador F., Martin A., López-Menéndez C., Moreno-Bueno G., Santos V., Vázquez-Naharro A., Santamaria P.G., Morales S., Dubus P., Muinelo-Romay L. (2017). Lysyl Oxidase-like Protein LOXL2 Promotes Lung Metastasis of Breast Cancer. Cancer Res..

[66] Shanbhag V.C., Gudekar N., Jasmer K., Papageorgiou C., Singh K., Petris M.J. (2021). Copper metabolism as a unique vulnerability in cancer. Biochim. Biophys. Acta Mol. Cell Res..

[67] Liburkin-Dan T., Toledano S., Neufeld G. (2022). Lysyl Oxidase Family Enzymes and Their Role in Tumor Progression. Int. J. Mol. Sci..

[68] Xiao Q., Ge G. (2012). Lysyl oxidase, extracellular matrix remodeling and cancer metastasis. Cancer Microenviron..

[69] Baker A.M., Bird D., Lang G., Cox T.R., Erler J.T. (2013). Lysyl oxidase enzymatic function increases stiffness to drive colorectal cancer progression through FAK. Oncogene.

[70] Baker A.-M., Cox T.R., Bird D., Lang G., Murray G.I., Sun X.-F., Southall S.M., Wilson J.R., Erler J.T. (2011). The role of lysyl oxidase in SRC-dependent proliferation and metastasis of colorectal cancer. J. Natl. Cancer Inst..

[71] Kalinina T., Güngör C., Thieltges S., Möller-Krull M., Penas E.M.M., Wicklein D., Streichert T., Schumacher U., Kalinin V., Simon R. (2010). Establishment and characterization of a new human pancreatic adenocarcinoma cell line with high metastatic potential to the lung. BMC Cancer.

[72] MacDonald G., Nalvarte I., Smirnova T., Vecchi M., Aceto N., Doelemeyer A., Frei A., Lienhard S., Wyckoff J., Hess D. (2014). Memo is a copper-dependent redox protein with an essential role in migration and metastasis. Sci. Signal..

[73] Labrecque C.L., Hilton C.N., Airas J., Blake A., Rubenstein K.J., Parish C.A., Pollock J.A. (2021). Identification of Phenazine-Based MEMO1 Small-Molecule Inhibitors: Virtual Screening, Fluorescence Polarization Validation, and Inhibition of Breast Cancer Migration. ChemMedChem.

[74] Ren X., Jing Y.X., Zhou Z.W., Yang J.W. (2022). Knockdown of circRNA-Memo1 Reduces Hypoxia/Reoxygenation Injury in Human Brain Endothelial Cells Through miRNA-17-5p/SOS1 Axis. Mol. Neurobiol..

[75] Xu K., Shi J., Mo D., Yang Y., Fu Q., Luo Y. (2020). miR-219a-1 inhibits colon cancer cells proliferation and invasion by targeting MEMO1. Cancer Biol. Ther..

[76] Chen G., Huynh M., Fehrenbacher L., West H., Lara P.N., Yavorkovsky L.L., Russin M., Goldstein D., Gandara D., Lau D. (2009). Phase II trial of irinotecan and carboplatin for extensive or relapsed small-cell lung cancer. J. Clin. Oncol..

[77] Galsky M.D., Chen G.J., Oh W.K., Bellmunt J., Roth B.J., Petrioli R., Dogliotti L., Dreicer R., Sonpavde G., Galsky M.D. (2012). Comparative effectiveness of cisplatin-based and carboplatin-based chemotherapy for treatment of advanced urothelial carcinoma. Ann. Oncol..

[78] Jr G.W.S., Sr P.J.L., Roth B.J., Einhorn L.H. (1988). Cisplatin as first-line therapy for metastatic breast cancer. J. Clin. Oncol..

[79] Khan A.B., D’Souza B.J., Wharam M.D., Champion L.A., Sinks L.F., Woo S.Y., Mccullough D., Leventhal B. (1982). Cisplatin therapy in recurrent childhood brain tumors. Cancer Treat. Rep..

[80] Mandala M., Ferretti G., Barni S. (2004). Oxaliplatin in colon cancer. N. Engl. J. Med..

[81] McGuire W.P., Hoskins W.J., Brady M.F., Kucera P.R., Partridge E.E., Look K.Y., Clarke-Pearson D.L., Davidson M. (1996). Cyclophosphamide and cisplatin compared with paclitaxel and cisplatin in patients with stage III and stage IV ovarian cancer. N. Engl. J. Med..

[82] Chen X., Wu Y., Dong H., Zhang C.Y., Zhang Y. (2013). Platinum-based agents for individualized cancer treatment. Curr. Mol. Med..

[83] Kuo M.T., Chen H.H., Song I.S., Savaraj N., Ishikawa T. (2007). The roles of copper transporters in cisplatin resistance. Cancer Metastasis Rev..

[84] Kalayda G.V., Wagner C.H., Jaehde U. (2012). Relevance of copper transporter 1 for cisplatin resistance in human ovarian carcinoma cells. J. Inorg. Biochem..

[85] Lee Y.-Y., Choi C.H., Do I.-G., Song S.Y., Lee W., Park H.S., Song T.J., Kim M.K., Kim T.-J., Lee J.-W. (2011). Prognostic value of the copper transporters, CTR1 and CTR2, in patients with ovarian carcinoma receiving platinum-based chemotherapy. Gynecol. Oncol..

[86] Li Y.Q., Yin J.Y., Liu Z.Q., Li X.P. (2018). Copper efflux transporters ATP7A and ATP7B: Novel biomarkers for platinum drug resistance and targets for therapy. IUBMB Life.

[87] Mangala L.S., Zuzel V., Schmandt R., Leshane E.S., Halder J.B., Armaiz-Pena G.N., Spannuth W.A., Tanaka T., Shahzad M.M., Lin Y.G. (2009). Therapeutic Targeting of ATP7B in Ovarian Carcinoma. Clin. Cancer Res..

[88] Xu W., Cai B., Chen J., Li L., Zhang J., Sun Y., Wan X. (2008). ATP7B antisense oligodeoxynucleotides increase the cisplatin sensitivity of human ovarian cancer cell line SKOV3ipl. Int. J. Gynecol. Cancer.

[89] Yu Z., Cao W., Ren Y., Zhang Q., Liu J. (2020). ATPase copper transporter A, negatively regulated by miR-148a-3p, contributes to cisplatin resistance in breast cancer cells. Clin. Transl. Med..

[90] Oliveri V. (2022). Selective Targeting of Cancer Cells by Copper Ionophores: An Overview. Front. Mol. Biosci..

[91] Kannappan V., Ali M., Small B., Rajendran G., Elzhenni S., Taj H., Wang W., Dou Q.P. (2021). Recent Advances in Repurposing Disulfiram and Disulfiram Derivatives as Copper-Dependent Anticancer Agents. Front. Mol. Biosci..

[92] Wehbe M., Anantha M., Backstrom I., Leung A., Chen K., Malhotra A., Edwards K., Bally M.B. (2016). Nanoscale Reaction Vessels Designed for Synthesis of Copper-Drug Complexes Suitable for Preclinical Development. PLoS ONE.

[93] Wehbe M., Anantha M., Shi M., Leung A.W.-Y., Dragowska W.H., Sanche L., Bally M.B. (2017). Development and optimization of an injectable formulation of copper diethyldithiocarbamate, an active anticancer agent. Int. J. Nanomed..

[94] Noll C.A., Betz L.D. (1952). Determination of Copper Ion by Modified Sodium Diethyldithiocarbamate Procedure. Anal. Chem..

[95] Johansson B. (1992). A review of the pharmacokinetics and pharmacodynamics of disulfiram and its metabolites. Acta Psychiatr. Scand..

[96] Ekinci E., Rohondia S., Khan R., Dou Q.P. (2019). Repurposing Disulfiram as An Anti-Cancer Agent: Updated Review on Literature and Patents. Recent. Pat. Anticancer. Drug Discov..

[97] Ning X., Chen X., Li R., Li Y., Lin Z., Yin Y. (2025). Identification of a novel cuproptosis inducer that induces ER stress and oxidative stress to trigger immunogenic cell death in tumors. Free Radic. Biol. Med..

[98] Ling Y.-Y., Shen Q.-H., Hao L., Li Z.-Y., Yu L.-B., Chen X.-X., Tan C.-P. (2025). Theranostic Rhenium Complexes as Suborganelle-Targeted Copper Ionophores To Stimulate Cuproptosis for Cancer Immunotherapy. ACS Appl. Mater. Interfaces.

[99] Baldari S., Di Rocco G., Toietta G. (2020). Current Biomedical Use of Copper Chelation Therapy. Int. J. Mol. Sci..

[100] Lowndes S.A., Harris A.L. (2004). Copper chelation as an antiangiogenic therapy. Oncol. Res..

[101] Steinbrueck A., Sedgwick A.C., Brewster J.T., Yan K.-C., Shang Y., Knoll D.M., Vargas-Zúñiga G.I., He X.-P., Tian H., Sessler J.L. (2020). Transition metal chelators, pro-chelators, and ionophores as small molecule cancer chemotherapeutic agents. Chem. Soc. Rev..

[102] Morisawa A., Okui T., Shimo T., Ibaragi S., Okusha Y., Ono M., Nguyen T.T.H., Hassan N.M.M., Sasaki A. (2018). Ammonium tetrathiomolybdate enhances the antitumor effects of cetuximab via the suppression of osteoclastogenesis in head and neck squamous carcinoma. Int. J. Oncol..

[103] Zhou P., Qin J., Zhou C., Wan G., Liu Y., Zhang M., Yang X., Zhang N., Wang Y. (2019). Multifunctional nanoparticles based on a polymeric copper chelator for combination treatment of metastatic breast cancer. Biomaterials.

[104] Yoo J.Y., Pradarelli J., Haseley A., Wojton J., Kaka A., Bratasz A., Alvarez-Breckenridge C.A., Yu J.-G., Powell K., Mazar A.P. (2012). Copper Chelation Enhances Antitumor Efficacy and Systemic Delivery of Oncolytic HSV. Clin. Cancer Res..

[105] Wang W., McLeod H.L., Cassidy J. (2003). Disulfiram-mediated inhibition of NF-?B activity enhances cytotoxicity of 5-fluorouracil in human colorectal cancer cell lines. Int. J. Cancer.

[106] Skrott Z., Majera D., Gursky J., Buchtova T., Hajduch M., Mistrik M., Bartek J. (2019). Disulfiram’s anti-cancer activity reflects targeting NPL4, not inhibition of aldehyde dehydrogenase. Oncogene.

[107] Shah O’Brien P., Xi Y., Miller J.R., Brownell A.L., Zeng Q., Yoo G.H., Danielle M., Garshott D.M., O’Brien M.B., Galinato A.B. (2019). Disulfiram (Antabuse) activates ROS-dependent ER stress and apoptosis in oral cavity squamous cell carcinoma. J. Clin. Med..

[108] Daniel K.G., Chen D., Orlu S., Cui Q.C., Miller F.R., Dou Q.P. (2005). Clioquinol and pyrrolidine dithiocarbamate complex with copper to form proteasome inhibitors and apoptosis inducers in human breast cancer cells. Breast Cancer Res..

[109] Chen S.-H., Lin J.-K., Liang Y.-C., Pan M.-H., Liu S.-H., Lin-Shiau S.-Y. (2008). Involvement of activating transcription factors JNK, NF-κB, and AP-1 in apoptosis induced by pyrrolidine dithiocarbamate/Cu complex. Eur. J. Pharmacol..

[110] Németh Z.H., Haskó G., Vizi E.S. (1998). Pyrrolidine Dithiocarbamate Augments IL-10, Inhibits TNF-α, MIP-1α, IL-12, and Nitric Oxide Production and Protects From the Lethal Effect of Endotoxin. Shock.

[111] Zheng P., Zhou C., Lu L., Liu B., Ding Y. (2022). Elesclomol: A copper ionophore targeting mitochondrial metabolism for cancer therapy. J. Exp. Clin. Cancer Res..

[112] Gupte A., Mumper R.J. (2007). Copper chelation by D-penicillamine generates reactive oxygen species that are cytotoxic to human leukemia and breast cancer cells. Free Radic. Biol. Med..

[113] Pitman S.K., Huynh T., Bjarnason T.A., An J., Malkhasyan K.A. (2019). A case report and focused literature review of d-penicillamine and severe neutropenia: A serious toxicity from a seldom-used drug. Clin. Case Rep..

[114] Wadhwa S., Mumper R.J. (2013). D-penicillamine and other low molecular weight thiols: Review of anticancer effects and related mechanisms. Cancer Lett..

[115] Liu Y.L., Bager C.L., Willumsen N., Ramchandani D., Kornhauser N., Ling L., Cobham M., Andreopoulou E., Cigler T., Moore A. (2021). Tetrathiomolybdate (TM)-associated copper depletion influences collagen remodeling and immune response in the pre-metastatic niche of breast cancer. NPJ Breast Cancer.

[116] Hou G., Abrams G.D., Dick R., Brewer G.J. (2008). Efficacy of tetrathiomolybdate in a mouse model of multiple sclerosis. Transl. Res..

[117] Yoshii J., Yoshiji H., Kuriyama S., Ikenaka Y., Noguchi R., Okuda H., Tsujinoue H., Nakatani T., Kishida H., Nakae D. (2001). The copper-chelating agent, trientine, suppresses tumor development and angiogenesis in the murine hepatocellular carcinoma cells. Int. J. Cancer.

[118] Ismail N.I., Othman I., Abas F., Lajis N.H., Naidu R. (2019). Mechanism of Apoptosis Induced by Curcumin in Colorectal Cancer. Int. J. Mol. Sci..

[119] Jagetia G.C., Aggarwal B.B. (2007). “Spicing Up” of the Immune System by Curcumin. J. Clin. Immunol..

[120] White C., Lee J., Kambe T., Fritsche K., Petris M.J. (2009). A Role for the ATP7A Copper-transporting ATPase in Macrophage Bactericidal Activity. J. Biol. Chem..

[121] Elzanowska H., Wolcott R.G., Hannum D.M., Hurst J.K. (1995). Bactericidal properties of hydrogen peroxide and copper or iron-containing complex ions in relation to leukocyte function. Free Radic. Biol. Med..

[122] Toebak M., Pohlmann P., Sampat-Sardjoepersad S., von Blomberg B., Bruynzeel D., Scheper R., Rustemeyer T., Gibbs S. (2006). CXCL8 secretion by dendritic cells predicts contact allergens from irritants. Toxicol. Vitr..

[123] Pocino M. (1991). Influence of the oral administration of excess copper on the immune response. Fundam. Appl. Toxicol..

[124] Turnlund J.R., A Jacob R., Keen C.L., Strain J., Kelley D.S., Domek J.M., Keyes W.R., Ensunsa J.L., Lykkesfeldt J., Coulter J. (2004). Long-term high copper intake: Effects on indexes of copper status, antioxidant status, and immune function in young men. Am. J. Clin. Nutr..

[125] Członkowska A., Milewski B. (1976). Immunological observations on patients with Wilson’s disease. J. Neurol. Sci..

[126] Soderberg L.S.F., Barnett J.B., Sorenson J.R.J. (1989). Copper Complexes Stimulate Hemopoiesis and Lymphopoiesis. Copper Bioavailability and Metabolism.

[127] Percival S.S. (1998). Copper and immunity. Am. J. Clin. Nutr..

[128] Stabel J.R., Spears J.W. (1989). Effect of Copper on Immune Function and Disease Resistance. Copper Bioavailability and Metabolism.

[129] Percival S.S. (1995). Neutropenia caused by copper deficiency: Possible mechanisms of action. Nutr. Rev..

[130] Kerr J.F.R., Wyllie A.H., Currie A.R. (1972). Apoptosis: A Basic Biological Phenomenon with Wideranging Implications in Tissue Kinetics. Br. J. Cancer.

[131] Koller L.D., Mulhern S.A., Frankel N.C., Steven M.G., Williams J.R. (1987). Immune dysfunction in rats fed a diet deficient in copper. Am. J. Clin. Nutr..

[132] Lukasewycz O.A., Prohaska J.R., Meyer S.G., Schmidtke J.R., Hatfield S.M., Marder P. (1985). Alterations in lymphocyte subpopulations in copper-deficient mice. Infect. Immun..

[133] Bonham M., O’Connor J.M., Hannigan B.M., Strain J.J. (2002). The immune system as a physiological indicator of marginal copper status?. Br. J. Nutr..

[134] Galluzzi L., Vitale I., Aaronson S.A., Abrams J.M., Adam D., Agostinis P., Alnemri E.S., Altucci L., Amelio I., Andrews D.W. (2018). Molecular mechanisms of cell death: Recommendations of the Nomenclature Committee on Cell Death 2018. Cell Death Differ..

[135] Casares N., Pequignot M.O., Tesniere A., Ghiringhelli F., Roux S., Chaput N., Schmitt E., Hamai A., Hervas-Stubbs S., Obeid M. (2005). Caspase-dependent immunogenicity of doxorubicin-induced tumor cell death. J. Exp. Med..

[136] Mole R.H. (1953). Whole body irradiation; radiobiology or medicine?. Br. J. Radiol..

[137] Craig D.J., Nanavaty N.S., Devanaboyina M., Stanbery L., Hamouda D., Edelman G., Dworkin L., Nemunaitis J.J. (2021). The abscopal effect of radiation therapy. Future Oncol..

[138] Galluzzi L., Vitale I., Warren S., Adjemian S., Agostinis P., Martinez A.B., Chan T.A., Coukos G., Demaria S., Deutsch E. (2020). Consensus guidelines for the definition, detection and interpretation of immunogenic cell death. J. Immunother. Cancer.

[139] Kepp O., Senovilla L., Vitale I., Vacchelli E., Adjemian S., Agostinis P., Apetoh L., Aranda F., Barnaba V., Bloy N. (2014). Consensus guidelines for the detection of immunogenic cell death. Oncoimmunology.

[140] Obeid M., Tesniere A., Ghiringhelli F., Fimia G.M., Apetoh L., Perfettini J.-L., Castedo M., Mignot G., Panaretakis T., Casares N. (2007). Calreticulin exposure dictates the immunogenicity of cancer cell death. Nat. Med..

[141] Zhou J., Wang G., Chen Y., Wang H., Hua Y., Cai Z. (2019). Immunogenic cell death in cancer therapy: Present and emerging inducers. J. Cell. Mol. Med..

[142] Zitvogel L., Kepp O., Senovilla L., Menger L., Chaput N., Kroemer G. (2010). Immunogenic Tumor Cell Death for Optimal Anticancer Therapy: The Calreticulin Exposure Pathway. Clin. Cancer Res..

[143] Panaretakis T., Kepp O., Brockmeier U., Tesniere A., Bjorklund A., Chapman D.C., Durchschlag M., Joza N., Pierron G., van Endert P. (2009). Mechanisms of pre-apoptotic calreticulin exposure in immunogenic cell death. EMBO J..

[144] Galluzzi L., Humeau J., Buqué A., Zitvogel L., Kroemer G. (2020). Immunostimulation with chemotherapy in the era of immune checkpoint inhibitors. Nat. Rev. Clin. Oncol..

[145] Ye W., Gunti S., Allen C.T., Hong Y., Clavijo P.E., Van Waes C., Schmitt N.C. (2020). ASTX660, an antagonist of cIAP1/2 and XIAP, increases antigen processing machinery and can enhance radiation-induced immunogenic cell death in preclinical models of head and neck cancer. Oncoimmunology.

[146] Deutsch E., Chargari C., Galluzzi L., Kroemer G. (2019). Optimising efficacy and reducing toxicity of anticancer radioimmunotherapy. Lancet Oncol..

[147] Kepp O., Zitvogel L., Kroemer G. (2019). Clinical evidence that immunogenic cell death sensitizes to PD-1/PD-L1 blockade. Oncoimmunology.

[148] Yu Z., Guo J., Hu M., Gao Y., Huang L. (2020). Icaritin Exacerbates Mitophagy and Synergizes with Doxorubicin to Induce Immunogenic Cell Death in Hepatocellular Carcinoma. ACS Nano.

[149] Zhu H., Shan Y., Ge K., Lu J., Kong W., Jia C. (2020). Oxaliplatin induces immunogenic cell death in hepatocellular carcinoma cells and synergizes with immune checkpoint blockade therapy. Cell. Oncol..

[150] Powles T., Valderrama B.P., Gupta S., Bedke J., Kikuchi E., Hoffman-Censits J., Iyer G., Vulsteke C., Park S.H., Shin S.J. (2024). Enfortumab Vedotin and Pembrolizumab in Untreated Advanced Urothelial Cancer. N. Engl. J. Med..

[151] van der Heijden M.S., Sonpavde G., Powles T., Necchi A., Burotto M., Schenker M., Sade J.P., Bamias A., Beuzeboc P., Bedke J. (2023). Nivolumab plus Gemcitabine–Cisplatin in Advanced Urothelial Carcinoma. N. Engl. J. Med..

[152] Fucikova J., Kepp O., Kasikova L., Petroni G., Yamazaki T., Liu P., Zhao L., Spisek R., Kroemer G., Galluzzi L. (2020). Detection of immunogenic cell death and its relevance for cancer therapy. Cell Death Dis..

[153] Vacchelli E., Galluzzi L., Fridman W.H., Galon J., Sautès-Fridman C., Tartour E., Kroemer G. (2012). Trial watch: Chemotherapy with immunogenic cell death inducers. Oncoimmunology.

[154] Vacchelli E., Senovilla L., Eggermont A., Fridman W.H., Galon J., Zitvogel L., Kroemer G., Galluzzi L. (2013). Trial watch: Chemotherapy with immunogenic cell death inducers. Oncoimmunology.

[155] Vacchelli E., Aranda F., Eggermont A., Galon J., Sautès-Fridman C., Cremer I., Zitvogel L., Kroemer G., Galluzzi L. (2014). Trial watch: Chemotherapy with immunogenic cell death inducers. Oncoimmunology.

[156] Pol J., Vacchelli E., Aranda F., Castoldi F., Eggermont A., Cremer I., Sautès-Fridman C., Fucikova J., Galon J., Spisek R. (2015). Trial Watch: Immunogenic cell death inducers for anticancer chemotherapy. Oncoimmunology.

[157] Garg A.D., More S., Rufo N., Mece O., Sassano M.L., Agostinis P., Zitvogel L., Kroemer G., Galluzzi L. (2017). Trial watch: Immunogenic cell death induction by anticancer chemotherapeutics. Oncoimmunology.

[158] Vanmeerbeek I., Sprooten J., De Ruysscher D., Tejpar S., Vandenberghe P., Fucikova J., Spisek R., Zitvogel L., Kroemer G., Galluzzi L. (2020). Trial watch: Chemotherapy-induced immunogenic cell death in immuno-oncology. Oncoimmunology.

[159] Vo T.T.T., Peng T.-Y., Nguyen T.H., Bui T.N.H., Wang C.-S., Lee W.-J., Chen Y.-L., Wu Y.-C., Lee I.-T. (2024). The crosstalk between copper-induced oxidative stress and cuproptosis: A novel potential anticancer paradigm. Cell Commun. Signal..

[160] Maharjan R., Choi J.U., Kweon S., Pangeni R., Lee N.K., Park S.J., Chang K.-Y., Park J.W., Byun Y. (2022). A novel oral metronomic chemotherapy provokes tumor specific immunity resulting in colon cancer eradication in combination with anti-PD-1 therapy. Biomaterials.

[161] Shitara K., Van Cutsem E., Bang Y.-J., Fuchs C., Wyrwicz L., Lee K.-W., Kudaba I., Garrido M., Chung H.C., Lee J. (2020). Efficacy and Safety of Pembrolizumab or Pembrolizumab Plus Chemotherapy vs Chemotherapy Alone for Patients With First-line, Advanced Gastric Cancer. JAMA Oncol..

[162] Janjigian Y.Y., Shitara K., Moehler M., Garrido M., Salman P., Shen L., Wyrwicz L., Yamaguchi K., Skoczylas T., Campos Bragagnoli A. (2021). First-line nivolumab plus chemotherapy versus chemotherapy alone for advanced gastric, gastro-oesophageal junction, and oesophageal adenocarcinoma (CheckMate 649): A randomised, open-label, phase 3 trial. Lancet.

[163] Liu P., Chen J., Zhao L., Hollebecque A., Kepp O., Zitvogel L., Kroemer G. (2022). PD-1 blockade synergizes with oxaliplatin-based, but not cisplatin-based, chemotherapy of gastric cancer. Oncoimmunology.

[164] Arai H., Xiao Y., Loupakis F., Kawanishi N., Wang J., Battaglin F., Soni S., Zhang W., Mancao C., Salhia B. (2020). Immunogenic cell death pathway polymorphisms for predicting oxaliplatin efficacy in metastatic colorectal cancer. J. Immunother. Cancer.

[165] Skrott Z., Cvek B. (2012). Diethyldithiocarbamate complex with copper: The mechanism of action in cancer cells. Mini-Rev. Med. Chem..

[166] Skrott Z., Mistrik M., Andersen K.K., Friis S., Majera D., Gursky J., Ozdian T., Bartkova J., Turi Z., Moudry P. (2017). Alcohol-abuse drug disulfiram targets cancer via p97 segregase adaptor NPL4. Nature.

[167] Guo W., Jia L., Xie L., Kiang J.G., Wang Y., Sun F., Lin Z., Wang E., Zhang Y., Huang P. (2024). Turning anecdotal irradiation-induced anticancer immune responses into reproducible in situ cancer vaccines via disulfiram/copper-mediated enhanced immunogenic cell death of breast cancer cells. Cell Death Dis..

[168] Kuemmel S., Gluz O., Reinisch M., Kostara A., Scheffen I., Graeser M., Wuerstlein R., Nitz U., Luedtke-Heckenkamp K., Hartkopf A. (2022). Abstract PD10-11: Keyriched-1—A prospective, multicenter, open label, neoadjuvant phase ii single arm study with pembrolizumab in combination with dual anti-HER2 blockade with trastuzumab and pertuzumab in early breast cancer patients with molecular HER2-enriched intrinsic subtype. Cancer Res..

[169] Loi S., Giobbie-Hurder A., Gombos A., Bachelot T., Hui R., Curigliano G., Campone M., Biganzoli L., Bonnefoi H., Jerusalem G. (2019). Pembrolizumab plus trastuzumab in trastuzumab-resistant, advanced, HER2-positive breast cancer (PANACEA): A single-arm, multicentre, phase 1b–2 trial. Lancet Oncol..

[170] Schmid P., Rugo H.S., Adams S., Schneeweiss A., Barrios C.H., Iwata H., Diéras V., Henschel V., Molinero L., Chui S.Y. (2020). Atezolizumab plus nab-paclitaxel as first-line treatment for unresectable, locally advanced or metastatic triple-negative breast cancer (IMpassion130): Updated efficacy results from a randomised, double-blind, placebo-controlled, phase 3 trial. Lancet Oncol..

[171] Schmid P., Cortes J., Dent R., Pusztai L., McArthur H., Kümmel S., Bergh J., Denkert C., Park Y.H., Hui R. (2022). Event-free Survival with Pembrolizumab in Early Triple-Negative Breast Cancer. N. Engl. J. Med..

[172] Cortes J., Cescon D.W., Rugo H.S., Nowecki Z., Im S.-A., Yusof M.M., Gallardo C., Lipatov O., Barrios C.H., Holgado E. (2020). Pembrolizumab plus chemotherapy versus placebo plus chemotherapy for previously untreated locally recurrent inoperable or metastatic triple-negative breast cancer (KEYNOTE-355): A randomised, placebo-controlled, double-blind, phase 3 clinical trial. Lancet.

[173] McDermott S.P., Wicha M.S. (2010). Targeting breast cancer stem cells. Mol. Oncol..

[174] Lei M.M.L., Lee T.K.W. (2021). Cancer Stem Cells: Emerging Key Players in Immune Evasion of Cancers. Front. Cell Dev. Biol..

[175] Demaria S., Ng B., Devitt M.L., Babb J.S., Kawashima N., Liebes L., Formenti S.C. (2004). Ionizing radiation inhibition of distant untreated tumors (abscopal effect) is immune mediated. Int. J. Radiat. Oncol. Biol. Phys..

[176] Dewan M.Z., Galloway A.E., Kawashima N., Dewyngaert J.K., Babb J.S., Formenti S.C., Demaria S. (2009). Fractionated but Not Single-Dose Radiotherapy Induces an Immune-Mediated Abscopal Effect when Combined with Anti–CTLA-4 Antibody. Clin. Cancer Res..

[177] Ni Y.-L., Chien P.-J., Hsieh H.-C., Shen H.-T., Lee H.-T., Chen S.-M., Chang W.-W. (2022). Disulfiram/Copper Suppresses Cancer Stem Cell Activity in Differentiated Thyroid Cancer Cells by Inhibiting BMI1 Expression. Int. J. Mol. Sci..

[178] Llovet J.M., Castet F., Heikenwalder M., Maini M.K., Mazzaferro V., Pinato D.J., Pikarsky E., Zhu A.X., Finn R.S. (2022). Immunotherapies for hepatocellular carcinoma. Nat. Rev. Clin. Oncol..

[179] Sangro B., Sarobe P., Hervás-Stubbs S., Melero I. (2021). Advances in immunotherapy for hepatocellular carcinoma. Nat. Rev. Gastroenterol. Hepatol..

[180] Bonaventura P., Shekarian T., Alcazer V., Valladeau-Guilemond J., Valsesia-Wittmann S., Amigorena S., Caux C., Depil S. (2019). Cold Tumors: A Therapeutic Challenge for Immunotherapy. Front. Immunol..

[181] Chiba T., Suzuki E., Yuki K., Zen Y., Oshima M., Miyagi S., Saraya A., Koide S., Motoyama T., Ogasawara S. (2014). Disulfiram Eradicates Tumor-Initiating Hepatocellular Carcinoma Cells in ROS-p38 MAPK Pathway-Dependent and -Independent Manners. PLoS ONE.

[182] Kaur P., Johnson A., Northcote-Smith J., Lu C., Suntharalingam K. (2020). Immunogenic Cell Death of Breast Cancer Stem Cells Induced by an Endoplasmic Reticulum-Targeting Copper (II) Complex. ChemBioChem.

[183] Han Y., Liu D., Li L. (2020). PD-1/PD-L1 pathway: Current researches in cancer. Am. J. Cancer Res..

[184] Chang E., Pelosof L., Lemery S., Gong Y., Goldberg K.B., Farrell A.T., Keegan P., Veeraraghavan J., Wei G., Blumenthal G.M. (2021). Systematic Review of PD-1/PD-L1 Inhibitors in Oncology: From Personalized Medicine to Public Health. Oncologist.

[185] Martins F., Sofiya L., Sykiotis G.P., Lamine F., Maillard M., Fraga M., Shabafrouz K., Ribi C., Cairoli A., Guex-Crosier Y. (2019). Adverse effects of immune-checkpoint inhibitors: Epidemiology, management and surveillance. Nat. Rev. Clin. Oncol..

[186] Doroshow D.B., Bhalla S., Beasley M.B., Sholl L.M., Kerr K.M., Gnjatic S., Wistuba I.I., Rimm D.L., Tsao M.S., Hirsch F.R. (2021). PD-L1 as a biomarker of response to immune-checkpoint inhibitors. Nat. Rev. Clin. Oncol..

[187] Davis A.A., Patel V.G. (2019). The role of PD-L1 expression as a predictive biomarker: An analysis of all US Food and Drug Administration (FDA) approvals of immune checkpoint inhibitors. J. Immunother. Cancer.

[188] Koirala P., Roth M.E., Gill J., Piperdi S., Chinai J.M., Geller D.S., Hoang B.H., Park A., Fremed M.A., Zang X. (2016). Immune infiltration and PD-L1 expression in the tumor microenvironment are prognostic in osteosarcoma. Sci. Rep..

[189] Okita R., Maeda A., Shimizu K., Nojima Y., Saisho S., Nakata M. (2017). PD-L1 overexpression is partially regulated by EGFR/HER2 signaling and associated with poor prognosis in patients with non-small-cell lung cancer. Cancer Immunol. Immunother..

[190] Zhao Y., Shi F., Zhou Q., Li Y., Wu J., Wang R., Song Q. (2020). Prognostic significance of PD-L1 in advanced non-small cell lung carcinoma. Medicine.

[191] Cha J.-H., Chan L.-C., Li C.-W., Hsu J.L., Hung M.-C. (2019). Mechanisms Controlling PD-L1 Expression in Cancer. Mol. Cell.

[192] He F., Chang C., Liu B., Li Z., Li H., Cai N., Wang H.-H. (2019). Copper (II) Ions Activate Ligand-Independent Receptor Tyrosine Kinase (RTK) Signaling Pathway. Biomed. Res. Int..

[193] Renoux G., Renoux M., Lemarie E., Lavandier M., Greco J., Bardos P., Lang J.M., Boilletot A., Oberling F., Armand J. (1983). Sodium diethyldithiocarbamate (imuthiol) and cancer. Adv. Exp. Med. Biol..

[194] (2016). Disulfiram in Patients with Metastatic Melanoma. ClinicalTrials.gov Identifier: NCT00256230. NCT00256230.

[195] (2019). Disulfiram Plus Arsenic Trioxide in Patients with Metastatic Melanoma and at Least One Prior Systemic Therapy. ClinicalTrials.gov Identifier: NCT00571116. NCT00571116.

[196] Goswami M., Gui G., Dillon L.W., E Lindblad K., Thompson J., Valdez J., Kim D.-Y., Ghannam J.Y., A Oetjen K., Destefano C.B. (2022). Pembrolizumab and decitabine for refractory or relapsed acute myeloid leukemia. J. Immunother. Cancer.

[197] (2022). Study of Chidamide, Decitabine and Immune Checkpoint Inhibitors in R/R NHL and Advanced Solid Tumors. ClinicalTrials.gov Identifier: NCT05320640. NCT05320640.

[198] Wang Q., Zhu T., Miao N., Qu Y., Wang Z., Chao Y., Wang J., Wu W., Xu X., Xu C. (2022). Disulfiram bolsters T-cell anti-tumor immunity through direct activation of LCK-mediated TCR signaling. EMBO J..

[199] Clark D.W., Palle K. (2016). Aldehyde dehydrogenases in cancer stem cells: Potential as therapeutic targets. Ann. Transl. Med..

[200] Bazewicz C.G., Dinavahi S.S., Schell T.D., Robertson G.P. (2019). Aldehyde dehydrogenase in regulatory T-cell development, immunity and cancer. Immunology.

[201] Mizuno T., Suzuki N., Makino H., Furui T., Morii E., Aoki H., Kunisada T., Yano M., Kuji S., Hirashima Y. (2015). Cancer stem-like cells of ovarian clear cell carcinoma are enriched in the ALDH-high population associated with an accelerated scavenging system in reactive oxygen species. Gynecol. Oncol..

[202] Chen J., Xia Q., Jiang B., Chang W., Yuan W., Ma Z., Liu Z., Shu X. (2015). Prognostic Value of Cancer Stem Cell Marker ALDH1 Expression in Colorectal Cancer: A Systematic Review and Meta-Analysis. PLoS ONE.

[203] Panigoro S.S., Kurnia D., Kurnia A., Haryono S.J., Albar Z.A. (2020). ALDH1 Cancer Stem Cell Marker as a Prognostic Factor in Triple-Negative Breast Cancer. Int. J. Surg. Oncol..

[204] Wang Z., Mo Y., Tan Y., Wen Z., Dai Z., Zhang H., Zhang X., Feng S., Liang X., Song T. (2022). The ALDH Family Contributes to Immunocyte Infiltration, Proliferation and Epithelial-Mesenchymal Transformation in Glioma. Front. Immunol..

[205] Flores M.L., Franco E.H., Cousido L.F.S., Minguito-Carazo C., Guadarrama O.S., González L.L., Pascual M.E.V., de la Torre A.J.M., Palomo A.G., González A.L. (2022). Relationship between Aldehyde Dehydrogenase, PD-L1 and Tumor-Infiltrating Lymphocytes with Pathologic Response and Survival in Breast Cancer. Cancers.

[206] Zhang H., Xia Y., Wang F., Luo M., Yang K., Liang S., An S., Wu S., Yang C., Chen D. (2021). Aldehyde Dehydrogenase 2 Mediates Alcohol-Induced Colorectal Cancer Immune Escape through Stabilizing PD-L1 Expression. Adv. Sci..

[207] Almozyan S., Colak D., Mansour F., Alaiya A., Al-Harazi O., Qattan A., Al-Mohanna F., Al-Alwan M., Ghebeh H. (2017). PD-L1 promotes OCT4 and Nanog expression in breast cancer stem cells by sustaining PI3K/AKT pathway activation. Int. J. Cancer.

[208] Mandell J.B., Douglas N., Ukani V., Beumer J.H., Guo J., Payne J., Newman R., Mancinelli L., Intini G., Anderson C.J. (2022). ALDH1A1 Gene Expression and Cellular Copper Levels between Low and Highly Metastatic Osteosarcoma Provide a Case for Novel Repurposing with Disulfiram and Copper. Sarcoma.

[209] Jin N., Zhu X., Cheng F., Zhang L. (2018). Disulfiram/copper targets stem cell-like ALDH + population of multiple myeloma by inhibition of ALDH1A1 and Hedgehog pathway. J. Cell. Biochem..

[210] Liu X., Wang L., Cui W., Yuan X., Lin L., Cao Q., Wang N., Li Y., Guo W., Zhang X. (2016). Targeting ALDH1A1 by disulfiram/copper complex inhibits non-small cell lung cancer recurrence driven by ALDH-positive cancer stem cells. Oncotarget.

[211] Mays D.C., Nelson A.N., Fauq A.H., Shriver Z.H., Veverka K.A., Naylor S., Lipsky J.J. (1995). S-Methyl N,N-diethylthiocarbamate sulfone, a potential metabolite of disulfiram and potent inhibitor of low Km mitochondrial aldehyde dehydrogenase. Biochem. Pharmacol..

[212] Dinavahi S.S., Bazewicz C.G., Gowda R., Robertson G.P. (2019). Aldehyde Dehydrogenase Inhibitors for Cancer Therapeutics. Trends Pharmacol. Sci..

[213] Yang F., Liao J., Yu W., Pei R., Qiao N., Han Q., Hu L., Li Y., Guo J., Pan J. (2020). Copper induces oxidative stress with triggered NF-κB pathway leading to inflammatory responses in immune organs of chicken. Ecotoxicol. Environ. Saf..

[214] Zhao H., Wang Y., Shao Y., Liu J., Wang S., Xing M. (2018). Oxidative stress-induced skeletal muscle injury involves in NF-κB/p53-activated immunosuppression and apoptosis response in copper (II) or/and arsenite-exposed chicken. Chemosphere.

[215] Liu H., Guo H., Deng H., Cui H., Fang J., Zuo Z., Deng J., Li Y., Wang X., Zhao L. (2020). Copper induces hepatic inflammatory responses by activation of MAPKs and NF-κB signalling pathways in the mouse. Ecotoxicol. Environ. Saf..

[216] Wang Z., Zhang Y.-H., Guo C., Gao H.-L., Zhong M.-L., Huang T.-T., Liu N.-N., Guo R.-F., Lan T., Zhang W. (2018). Tetrathiomolybdate Treatment Leads to the Suppression of Inflammatory Responses through the TRAF6/NFκB Pathway in LPS-Stimulated BV-2 Microglia. Front. Aging Neurosci..

[217] Pan Q., Kleer C.G., Van Golen K.L., Irani J., Bottema K.M., Bias C., De Carvalho M., A Mesri E., Robins D.M., Dick R.D. (2002). Copper deficiency induced by tetrathiomolybdate suppresses tumor growth and angiogenesis. Cancer Res..

[218] Qi L., Xu Z., Jiang X., Hu C., Zou X. (2004). Preparation and antibacterial activity of chitosan nanoparticles. Carbohydr. Res..

[219] Dautremepuits C., Betoulle S., Paris-Palacios S., Vernet G. (2004). Immunology-related perturbations induced by copper and chitosan in carp (*Cyprinus carpio* L.). Arch. Environ. Contam. Toxicol..

[220] Mohammadhassan Z., Mohammadkhani R., Mohammadi A., Zaboli K.A., Kaboli S., Rahimi H., Nosrati H., Danafar H. (2022). Preparation of copper oxide nanoparticles coated with bovine serum albumin for delivery of methotrexate. J. Drug Deliv. Sci. Technol..

[221] Xia L., Tan S., Zhou Y., Lin J., Wang H., Oyang L., Tian Y., Liu L., Su M., Wang H. (2018). Role of the NFκB-signaling pathway in cancer. Onco Targets Ther..

[222] Wang D.J., Ratnam N.M., Byrd J.C., Guttridge D.C. (2014). NF-κB Functions in Tumor Initiation by Suppressing the Surveillance of Both Innate and Adaptive Immune Cells. Cell Rep..

[223] Lalle G., Twardowski J., Grinberg-Bleyer Y. (2021). NF-κB in Cancer Immunity: Friend or Foe?. Cells.

[224] Amato C.M., Hintzsche J.D., Wells K., Applegate A., Gorden N.T., Vorwald V.M., Tobin R.P., Nassar K., Shellman Y.G., Kim J. (2020). Pre-Treatment Mutational and Transcriptomic Landscape of Responding Metastatic Melanoma Patients to Anti-PD1 Immunotherapy. Cancers.

[225] Grasso C.S., Tsoi J., Onyshchenko M., Abril-Rodriguez G., Ross-Macdonald P., Wind-Rotolo M., Champhekar A., Medina E., Torrejon D.Y., Shin D.S. (2020). Conserved Interferon-γ Signaling Drives Clinical Response to Immune Checkpoint Blockade Therapy in Melanoma. Cancer Cell.

[226] Rasmi R.R., Sakthivel K.M., Guruvayoorappan C. (2020). NF-κB inhibitors in treatment and prevention of lung cancer. Biomed. Pharmacother..

[227] Hideshima T., Ikeda H., Chauhan D., Okawa Y., Raje N., Podar K., Mitsiades C., Munshi N.C., Richardson P.G., Carrasco R.D. (2009). Bortezomib induces canonical nuclear factor-κB activation in multiple myeloma cells. Blood.

[228] Sokolowska O., Rodziewicz-Lurzynska A., Pilch Z., Kedzierska H., Chlebowska-Tuz J., Sosnowska A., Szumera-Cieckiewicz A., Sokol K., Barankiewicz J., Salomon-Perzynski A. (2022). Immune checkpoint inhibition improves antimyeloma activity of bortezomib and STING agonist combination in Vk*MYC preclinical model. Clin. Exp. Med..

[229] (2022). Standard Doses of Bortezomib and Pembrolizumab with or Without Pelareorep for the Treatment of Relapsed or Refractory Multiple Myeloma, AMBUSH Trial. ClinicalTrials.gov Identifier: NCT05514990. NCT05514990.

[230] Bessho R., Matsubara K., Kubota M., Kuwakado K., Hirota H., Wakazono Y., Lin Y.W., Okuda A., Kawai M., Nishikomori R. (1994). Pyrrolidine dithiocarbamate, a potent inhibitor of nuclear factor κB (NF-κB) activation, prevents apoptosis in human promyelocytic leukemia HL-60 cells and thymocytes. Biochem. Pharmacol..

[231] Chung P.Y., Lam P.L., Zhou Y., Gasparello J., Finotti A., Chilin A., Marzaro G., Gambari R., Bian Z., Kwok W.M. (2018). Targeting DNA Binding for NF-κB as an Anticancer Approach in Hepatocellular Carcinoma. Cells.

[232] Zha J., Chen F., Dong H., Shi P., Yao Y., Zhang Y., Li R., Wang S., Li P., Wang W. (2014). Disulfiram targeting lymphoid malignant cell lines via ROS-JNK activation as well as Nrf2 and NF-κB pathway inhibition. J. Transl. Med..

[233] Xu B., Wang S., Li R., Chen K., He L., Deng M., Kannappan V., Zha J., Dong H., Wang W. (2017). Disulfiram/copper selectively eradicates AML leukemia stem cells in vitro and in vivo by simultaneous induction of ROS-JNK and inhibition of NF-κB and Nrf2. Cell Death Dis..

[234] Guo X., Xu B., Pandey S., Goessl E., Brown J., Armesilla A.L., Darling J.L., Wang W. (2010). Disulfiram/copper complex inhibiting NFκB activity and potentiating cytotoxic effect of gemcitabine on colon and breast cancer cell lines. Cancer Lett..

[235] Lee S.A. DISulfiram for COVID-19 (DISCO) Trial (DISCO) n.d.:ClinicalTrials.gov Identifier: NCT04485130. NCT04485130.

[236] Knights H.D.J. (2017). A Critical Review of the Evidence Concerning the HIV Latency Reversing Effect of Disulfiram, the Possible Explanations for Its Inability to Reduce the Size of the Latent Reservoir In Vivo, and the Caveats Associated with Its Use in Practice. AIDS Res. Treat..

[237] Ameh T., Sayes C.M. (2019). The potential exposure and hazards of copper nanoparticles: A review. Environ. Toxicol. Pharmacol..

[238] Mariadoss A.V.A., Saravanakumar K., Sathiyaseelan A., Venkatachalam K., Wang M.-H. (2020). Folic acid functionalized starch encapsulated green synthesized copper oxide nanoparticles for targeted drug delivery in breast cancer therapy. Int. J. Biol. Macromol..

[239] Naz S., Gul A., Zia M. (2020). Toxicity of copper oxide nanoparticles: A review study. IET Nanobiotechnol..

[240] Leung A.W.Y., Amador C., Wang L.C., Mody U.V., Bally M.B. (2019). What Drives Innovation: The Canadian Touch on Liposomal Therapeutics. Pharmaceutics.

[241] Krauss A.C., Gao X., Li L., Manning M.L., Patel P., Fu W., Janoria K.G., Gieser G., Bateman D.A., Przepiorka D. (2019). FDA Approval Summary: (Daunorubicin and Cytarabine) Liposome for Injection for the Treatment of Adults with High-Risk Acute Myeloid Leukemia. Clin. Cancer Res..

[242] Blair H.A. (2018). Daunorubicin/Cytarabine Liposome: A Review in Acute Myeloid Leukaemia. Drugs.

[243] Ramsay E.C., Anantha M., Zastre J., Meijs M., Zonderhuis J., Strutt D., Webb M.S., Waterhouse D., Bally M.B. (2008). Irinophore C: A Liposome Formulation of Irinotecan with Substantially Improved Therapeutic Efficacy against a Panel of Human Xenograft Tumors. Clin. Cancer Res..

[244] Ramsay E., Alnajim J., Anantha M., Taggar A., Thomas A., Edwards K., Karlsson G., Webb M., Bally M. (2006). Transition Metal-Mediated Liposomal Encapsulation of Irinotecan (CPT-11) Stabilizes the Drug in the Therapeutically Active Lactone Conformation. Pharm. Res..

[245] Patankar N., Anantha M., Ramsay E., Waterhouse D., Bally M. (2011). The Role of the Transition Metal Copper and the Ionophore A23187 in the Development of Irinophore CTM. Pharm. Res..

[246] Tardi P.G., Gallagher R.C., Johnstone S., Harasym N., Webb M., Bally M.B., Mayer L.D. (2007). Coencapsulation of irinotecan and floxuridine into low cholesterol-containing liposomes that coordinate drug release in vivo. Biochim. Et Biophys. Acta (BBA)-Biomembr..

[247] Dicko A., Tardi P., Xie X., Mayer L. (2007). Role of copper gluconate/triethanolamine in irinotecan encapsulation inside the liposomes. Int. J. Pharm..

[248] Szymański P., Frączek T., Markowicz M., Mikiciuk-Olasik E. (2012). Development of copper based drugs, radiopharmaceuticals and medical materials. Biometals.

[249] Wehbe M., Malhotra A.K., Anantha M., Lo C., Dragowska W.H., Dos Santos N., Bally M.B. (2018). Development of a copper-clioquinol formulation suitable for intravenous use. Drug Deliv. Transl. Res..

[250] Heroux D., Leung A.W.Y., Gilabert-Oriol R., Farzaneh S., Milne K., Wolf M., Alayoubi S., Singh H., MacFarlane T., Vito C. (2025). Immunogenic cell death in colorectal cancer models is modulated by baseline and ionophore-induced copper accumulation. Res. Sq..

[251] Heroux D., Sun X.X., Zhang S., Sharifiaghdam M., Leung A.W.Y., Farzaneh S., Milne K., MacFarlane T., Di Vito C., Nelson B.H. (2025). Copper ionophores drive divergent responses to immune checkpoint inhibition across colorectal tumor models.

[252] Li Y., Liu J., Chen Y., Weichselbaum R.R., Lin W. (2024). Nanoparticles Synergize Ferroptosis and Cuproptosis to Potentiate Cancer Immunotherapy. Adv. Sci..

[253] Wu H., Lu X., Hu Y., Baatarbolat J., Zhang Z., Liang Y., Zhang Y., Liu Y., Lv H., Jin X. (2025). Biomimic Nanodrugs Overcome Tumor Immunosuppressive Microenvironment to Enhance Cuproptosis/Chemodynamic-Induced Cancer Immunotherapy. Adv. Sci..

[254] Chen K., Zhou A., Zhou X., He J., Xu Y., Ning X. (2024). Cellular Trojan Horse initiates bimetallic Fe-Cu MOF-mediated synergistic cuproptosis and ferroptosis against malignancies. Sci. Adv..

[255] Jin X.-K., Liang J.-L., Zhang S.-M., Huang Q.-X., Zhang S.-K., Liu C.-J., Zhang X.-Z. (2023). Orchestrated copper-based nanoreactor for remodeling tumor microenvironment to amplify cuproptosis-mediated anti-tumor immunity in colorectal cancer. Mater. Today.

[256] Luo Y., Luo X., Ru Y., Zhou X., Liu D., Huang Q., Linghu M., Wu Y., Lv Z., Chen M. (2024). Copper(II)-Based Nano-Regulator Correlates Cuproptosis Burst and Sequential Immunogenic Cell Death for Synergistic Cancer Immunotherapy. Biomater. Res..

[257] Zhong X., Dai X., Wang Y., Wang H., Qian H., Wang X. (2022). Copper-based nanomaterials for cancer theranostics. WIREs Nanomed. Nanobiotechnol..

[258] Maryon E.B., Molloy S.A., Kaplan J.H. (2013). Cellular glutathione plays a key role in copper uptake mediated by human copper transporter 1. Am. J. Physiol. Cell Physiol..

[259] Ngamchuea K., Batchelor-McAuley C., Compton R.G. (2016). The Copper(II)-Catalyzed Oxidation of Glutathione. Chem.—A Eur. J..

[260] Yan L., Chang L., Tian Y., Hu J., Cao Z., Guo X., Geng B. (2025). Graphene Quantum Dot Sensitized Heterojunctions Induce Tumor-Specific Cuproptosis to Boost Sonodynamic and Chemodynamic Enhanced Cancer Immunotherapy. Adv. Sci..

[261] Wang P., Wang X., Ma L., Sahi S., Li L., Wang X., Wang Q., Chen Y., Chen W., Liu Q. (2018). Nanosonosensitization by Using Copper–Cysteamine Nanoparticles Augmented Sonodynamic Cancer Treatment. Part. Part. Syst. Charact..

[262] Hébert C.D., Elwell M.R., Travlos G.S., Fitz C.J., Bucher J.R. (1993). Subchronic Toxicity of Cupric Sulfate Administered in Drinking Water and Feed to Rats and Mice. Fundam. Appl. Toxicol..

[263] Molinaro C., Martoriati A., Pelinski L., Cailliau K. (2020). Copper Complexes as Anticancer Agents Targeting Topoisomerases I and II. Cancers.

[264] Hussain A., AlAjmi M.F., Rehman T., Amir S., Husain F.M., Alsalme A., Siddiqui M.A., AlKhedhairy A.A., Khan R.A. (2019). Copper(II) complexes as potential anticancer and Nonsteroidal anti-inflammatory agents: In vitro and in vivo studies. Sci. Rep..

[265] Guo W., Ye S., Cao N., Huang J., Gao J., Chen Q. (2010). ROS-mediated autophagy was involved in cancer cell death induced by novel copper(II) complex. Exp. Toxicol. Pathol..

[266] Bortolozzi R., Viola G., Porcù E., Consolaro F., Marzano C., Pellei M., Gandin V., Basso G. (2014). A novel copper(I) complex induces ER-stress-mediated apoptosis and sensitizes B-acute lymphoblastic leukemia cells to chemotherapeutic agents. Oncotarget.

[267] Lu X., Deng W., Wang S., Zhao S., Zhu B., Bai B., Mao Y., Lin J., Yi Y., Xie Z. (2024). PEGylated Elesclomol@Cu(II)-based Metal—organic framework with effective nanozyme performance and cuproptosis induction efficacy for enhanced PD-L1-based immunotherapy. Mater. Today Bio.

[268] Lu X., Chen X., Lin C., Yi Y., Zhao S., Zhu B., Deng W., Wang X., Xie Z., Rao S. (2024). Elesclomol Loaded Copper Oxide Nanoplatform Triggers Cuproptosis to Enhance Antitumor Immunotherapy. Adv. Sci..

[269] Heroux D., Leung A.W., Gilabert-Oriol R., Kulkarni J., Anantha M., Cullis P.R., Bally M.B. (2025). Liposomal delivery of a disulfiram metabolite drives copper-mediated tumor immunity. Int. J. Pharm..

[270] Yu Z., Cao L., Shen Y., Chen J., Li H., Li C., Yin J., Li Y., Meng Y., Li X. (2025). Inducing Cuproptosis with Copper Ion-Loaded Aloe Emodin Self-Assembled Nanoparticles for Enhanced Tumor Photodynamic Immunotherapy. Adv. Healthc. Mater..

[271] Wang Z., Li Y., Wang C., Lan J., Li J., Liu G., Chen Y., Yu D., Liu Z., Gao F. (2026). Disrupting intracellular redox homeostasis through copper-driven dual cell death to induce anti-tumor immunotherapy. Biomaterials.

[272] Jiang C., Li X., Wan S., Ji S., Wang Q., Hu S., Chen P., Wang B., Ge T., Zhang J. (2025). Copper-Doped Polydopamine Nanoparticles-Mediated GSH/GPX4-Depleted Ferroptosis and Cuproptosis Sensitizes Lung Tumor to Checkpoint Blockade Immunotherapy. Small.

